# *ERF022* impacts the induction of somatic embryogenesis in Arabidopsis through the ethylene-related pathway

**DOI:** 10.1007/s00425-014-2225-9

**Published:** 2014-12-23

**Authors:** Katarzyna Nowak, Barbara Wójcikowska, Małgorzata D. Gaj

**Affiliations:** Department of Genetics, University of Silesia, Jagiellonska 28, 40-032 Katowice, Poland

**Keywords:** Auxin, Ethylene, ETHYLENE RESPONSE FACTOR022, LEAFY COTYLEDON2, Somatic embryogenesis, *YUCCA* genes

## Abstract

**The** ***ERF022*** **gene was found to affect embryogenic transition in somatic cells in Arabidopsis via the ethylene-related pathway. The study provides evidence that** ***ERF022*** **-** ***LEC2*** **interaction is involved in the auxin–ethylene crosstalk that operates in somatic embryogenesis induction.**

The *ERF022* gene of the ERF family was previously identified among the transcription factor genes that were differentially expressed in an embryogenic culture of Arabidopsis. A strong inhibition of the gene was found to be associated with the induction of somatic embryogenesis (SE) and an *erf022* mutant was indicated to display a substantially impaired capacity for SE. Therefore, the molecular function of *ERF022* in the induction of SE was studied in the present work. A phenotype of an *erf022* mutant was indicated as being related to an increased content of ethylene. The results further suggest that the *ERF022* controls the genes that are involved in both the biosynthesis (*ACS7*) and signalling (*ERF1*, *ETR1*) of ethylene and indicate that the *ERF022* is a new regulatory element in ethylene-related responses that negatively control the ethylene content and perception. It is proposed that the negative impact of ethylene on the induction of SE may result from a modulation of the auxin-related genes that control the embryogenic transition in somatic cells. Among them, the *LEC2*, which is a key regulator of the induction of SE through the stimulation of auxin synthesis, was possibly related to *ERF022.* The results of the study provide new hormone-related clues to define the genetic network that governs SE. A putative model of the regulatory pathway is proposed that is involved in the induction of SE in which the auxin–ethylene interactions are controlled by *ERF022* and *LEC2* and their targets.

**Electronic supplementary material:**

The online version of this article (doi:10.1007/s00425-014-2225-9) contains supplementary material, which is available to authorized users.

## Introduction

Somatic embryogenesis (SE), a plant regeneration process in which embryos are derived from the somatic tissue, is being studied intensively in order to reveal the molecular mechanisms of plant cell totipotency. Significant progress in the identification of the genetic determinants of embryogenic development that are switched in somatic cells has been made since Arabidopsis was recommended as the model to identify SE-specific genes (Gaj 2004). Accordingly, several genes encoding transcription factors (TFs) that have an essential function in the induction of SE have been identified in Arabidopsis, including *BABY BOOM* (*BBM*, Boutilier et al. 2002), *WUSCHEL* (*WUS*, Zuo et al. 2002), *AGAMOUS*-*Like15* (*AGL15*, Harding et al. 2003), *LEAFY COTYLEDON* (*LEC*, Gaj et al. 2005), *LEC1*-*LIKE* (*L1L*, Yamamoto et al. 2009), *AtMYB115* and *AtMYB118* (Wang et al. 2009) and *EMBRYOMAKER* (*EMK*, Tsuwamoto et al. 2010). The key role of TF genes in the embryogenic reprogramming of somatic cells in plants was confirmed by the indication that an extensive modulation of over 700 TF genes accompanies the induction of SE in Arabidopsis (Gliwicka et al. 2013). Numerous TFs with SE-specific expression pattern were annotated to stress responses and members of the AP2/EREBP family and ERF subfamily related with ethylene were identified among them (Gliwicka et al. 2013). The involvement of several *AP2/ERF* genes in the induction of SE was also indicated in embryogenic cultures of other plants, including *Medicago truncatula* (Mantiri et al. 2008), *Triticum aestivum* (Singla et al. 2007), *Cichorium intybus* (Legrand et al. 2007), *Glycine max* (Thibaud-Nissen et al. 2003). It is possible that the SE-modulated transcription of *AP2/ERF* genes may reflect a general stress response inevitably associated with in vitro cultured tissues and induced by wounding or hormonal treatment (Zavattieri et al. 2010). However, some *AP2/ERF* genes, including *BBM* (Boutilier et al. 2002) and *EMK* (Tsuwamoto et al. 2010) were indicated to promote embryo development in Arabidopsis somatic tissues, which implies that they have a specific function in the induction of SE.

The representation of *ERF* genes in SE-transcriptome suggests the involvement of ethylene, which is a stress-related hormone in the mechanisms that operate during the induction of SE. Ethylene, a gaseous plant hormone, controls numerous developmental processes in plants (Chen et al. 2005; Zhu and Guo 2008) and its involvement in plant responses to abiotic and biotic stresses has been well documented (Chen et al. 2005; Ma et al. 2010). However, in contrast to auxin, which is widely recognized as a key hormone in the induction of SE (Jimenez 2005), the role of ethylene in the hormonal regulation of the embryogenic transition seems to be underestimated.

The effect of ethylene on the induction of SE is plant specific and the hormone was reported to negatively influence an embryogenic culture in various species including gymnosperm and angiosperm plants (Saly et al. 2002; Giridhar et al. 2004; Kong et al. 2012). In contrast, an increase of ethylene promoted the induction of SE in *Daucus carota* (Nissen 1994) and some legumes (Mantiri et al. 2008; Zheng et al. 2013). These various plant and culture system-specific effects of ethylene on SE complicate the understanding of its role in embryogenic induction. One of the best characterised ethylene-related genes that are essential for the induction of SE is *MtSERF1*, which encodes the SOMATIC EMBRYOGENESIS RELATED FACTOR1 protein of the AP2/EREBP superfamily and was found to positively control the embryogenic transition in *M. truncatula* (Mantiri et al. 2008). Recently, *At5g61590*, another member of the *AP2/ERF* in Arabidopsis and an ortholog of *MtSERF1*, was reported to be the direct target of AGL15, which is a positive regulator of SE (Zheng et al. 2013). Nonetheless, the ethylene-related genetic elements that are involved in the induction of SE remain mostly unknown.

The aim of the study was to perform a functional analysis of the *ERF022* gene of an SE-specific expression during the induction of SE (Gliwicka et al. 2013). A relation between *ERF022* and ethylene was hypothesised due to the presence of the AP2/EREBP domain in the encoded TF. *ERF022* is a member of the *ERF* subfamily that was identified within the AP2/ERF superfamily, which encodes numerous TFs that bind to DNA in the AP2/ERF domain and control a broad range of biological processes in plants (Nakano et al. 2006). The *ERF022* intron-less sequence of 721 bp was classified to the genes of the IIIa subgroup of the *ERF* subfamily indicated to control plant responses to abiotic stresses such as temperature, drought and salt treatment (McGrath et al. 2005; Nakano et al. 2006). The presented results confirmed the essential role of *ERF022* in SE and proposed an ethylene-related mechanism of the *ERF022* gene function in SE. Moreover, the results suggest the *LEC2*, which is a positive regulator of the SE that is involved in auxin biosynthesis, to be related to *ERF022*. The study reveals the new genetic components interacting in auxin–ethylene crosstalk that operates during the induction of SE.

## Materials and methods

### Plant material and growth conditions

Plants of *Arabidopsis thaliana* (L.) Heynh. Col-0 (WT) and two transgenic lines with different *ERF022* (At1g33760) expression levels were used, including: pER8-ERF022 with an induced the overexpression of *ERF022* (Gliwicka et al. 2013) and the knock-out *erf022* mutant (N591690). In addition, the insertional mutants in genes that are involved in ethylene biosynthesis (*acs7*-*1* (At4g26200)—N16570; *acs1*-*1* (At2g43750)—N16563; *eto1* (At3g51770)—N8060; *eto3* (At3g49700)—N3072) and signalling (*ein3* (At3g20770)—N502856; *ein4* (At3g04580)—N527079; *ers1* (At2g40940)—N674910; *etr1* (At1g66340)—N636291; *ctr1* (At5g03730)—N672594; *ein2* (At5g03280)—N647320; *ers2* (At1g04310)—N757174; *etr2* (At3g23150)—N657634) and 35S::ERF1 (At3g23240) (N6143) transgenic lines were studied. The *tan1*-*2* (At4g29860) and *lec2* (At1g28300) mutants were kindly provided by J.J. Harada (California University, Davies, CA, USA), while the *cbp20* (At5g44200) mutant was kindly provided by Z. Szweykowska-Kulinska from the Institute of Molecular Biology and Biotechnology, AMU, Poznań, Poland. With the exception of pER8-ERF022 (Gliwicka et al. 2013), the seeds of the genotypes that were analysed were purchased from NASC (The Nottingham Arabidopsis Stock Center). The seeds were sown in 42-mm-diameter Jiffy-7 peat pots (Jiffy) and plants were grown in a ‘walk-in’ type phytotron under controlled conditions: 22 °C, 16 h/8 h (light/dark) and a light intensity of 100 µmol photons m^−2^ s^−1^. Cultures that were grown in vitro were maintained in a controlled growth chamber at 22 °C, 16 h/8 h (light/dark) and a light intensity of 50 µmol photons m^−2^ s^−1^.

### Somatic embryogenesis induced in vitro

Immature zygotic embryos (IZEs) at the late cotyledonary stage were used as the explants for in vitro cultures. IZEs were excised from siliques at 10–12 days after pollination, sterilised with sodium hypochlorite (20 % commercial bleach) and washed thoroughly with sterile water. A standard protocol for Arabidopsis was used to induce SE (Gaj 2001). The IZEs were cultured on an E5 solid medium containing B5 salts and vitamins (Gamborg et al. 1968) and supplemented with 5 µM 2,4-D (2,4-dichlorophenoxyacetic acid, Sigma), 20 g L^−1^ sucrose and 8 g L^−1^ agar (Oxoid, Hampshire, UK). As a control, IZEs were cultured on an auxin-free E0 medium (E5 without 2,4-D) to promote the development of seedlings. The explant capacity for SE was evaluated in an IZE-derived culture that was induced for 21 days on an E5 medium and two parameters were calculated—SE efficiency, i.e. the percentage of explants that formed somatic embryos and SE productivity, i.e. the average number of somatic embryos produced by embryogenic explant.

### Shoot organogenesis

Shoot organogenesis (ORG), which is an alternative to the SE regeneration process, was induced in a culture of IZEs that was cultured for 7 days in a liquid callus induction medium (CIM) with 2.2 µM 2,4-D and 0.2 µM kinetin (Feldman and Marks 1986) and subsequently transferred to a solid shoot induction medium (SIM) supplemented with 0.5 µM naphthalene acetic acid (NAA) and 4.4 µM benzyl adenine (BAP) according to Kraut et al. (2011). The explant capacity for ORG was evaluated in a culture that was induced for 21 days on an SIM medium and two parameters were evaluated—the percentage of explants that developed at least one shoot (ORG efficiency) and the average number of shoots produced by the IZE explant (ORG productivity). All culture combinations were evaluated in three replicates and at least 30 explants (ten explants/Petri dish) were analysed per replicate.

### Seed-derived seedlings

Seeds were sterilised with sodium chloride (20 % commercial bleach or chlorine gas) and plated onto an MS medium (Murashige and Skoog 1962) with MS salts, 10 g L^−1^ sucrose and 8 g L^−1^ agar and supplemented with the analysed compounds. Plates were chilled at 4 °C in the dark for 4 days and then kept at 22 °C with a 16 h-light/8 h-dark cycle with light intensity of 50 µmol photons m^−2^ s^−1^.

### Triple response test

Etiolated seedlings of Arabidopsis mutants disturbed in ethylene biosynthesis or signalling display developmental defects called “a triple response” which include: (1) an inhibition of the hypocotyl and root elongation; (2) swelling of the hypocotyl and (3) an exaggerated tightening of the apical hook (Guzman and Ecker 1990). A phenotypic analysis was conducted on the seedlings that had been grown in the dark in order to identify ethylene-related genotypes. The plated seeds were grown in the dark at 4 °C for the first 4 days and then kept at 22 °C. The 3-day-old seedlings that had been grown in the dark were carefully inspected to search for any ethylene-related phenotypic changes. Moreover, the lengths of the hypocotyl and root and the presence of an exaggerated apical hook were evaluated.

### Ethylene modulators

To reveal the effect of ethylene on the induction of SE various modulators that affect the ethylene level and perception were used in an E5 medium that included 1-aminocyclopropane-1-carboxylic acid (ACC), a precursor of ethylene biosynthesis; CoCl_2_ and aminoethoxyvinylglycine (AVG), inhibitors of ethylene biosynthesis; AgNO_3_, an inhibitor of ethylene perception and KMnO_4_, an inactivator of exogenously accumulated ethylene. Ethylene modulators were added to the SE-induction medium at different concentrations, including: 1, 5, 10 µM of ACC; 1, 10 µM of CoCl_2_; 1, 10, 15 µM of AVG; 1, 10, 100 µM of AgNO_3_ and 250 mM of KMnO_4_. The effect of ethylene modulators on the embryogenic capacity of the IZE-derived cultures was evaluated.

### *ERF022* and jasmonic acid (JA)

To verify the relation between *ERF022* and JA, the seedlings of three genotypes (Col-0, *erf022* and pER8-ERF022), which differ in the gene expression level, were germinated on an MS medium supplemented with 10 µM of MeJA. The root length of 7-day-old seedlings was analysed.

### Stress response analysis

To evaluate the stress response of the genotypes that were tested, the germination rate of seeds grown on an MS medium supplemented with NaCl and mannitol was determined. The percentage of germinated seeds was scored in the presence of 0, 50, 150 and 200 mM of NaCl and 0, 100, 200, 300 and 400 mM of mannitol. Germination was defined as the emergence of the radicle and cotyledons through the seed coat. In each experimental combination 600–700 seeds were grown in three replicates for 4 days at 22 °C were evaluated.

### Ethylene content

The ethylene content of Col-0, the *erf022* knock-out mutant and pER8-ERF022 overexpression line was analysed in 3-day-old seedlings using the gas chromatography method. Two hundred and fifty seeds of each genotype were germinated in 50-mL Erlenmeyer flasks filled with 40 mL of an MS medium and closed with a rubber stopper. The seeds were grown in the dark for 4 days at 4 °C and then kept at 22 °C for 3 days after which the air was taken for analysis according to Guzman and Ecker (1990). Ethylene production was also evaluated in the IZE-derived cultures and embryogenic versus non-embryogenic cultures were compared. To do this, 30 IZE explants of Col-0 were cultured on an E5 medium for 15 days in standard SE-promoting conditions. The explants that developed somatic embryos (embryogenic culture) and those that failed in the induction of SE and produced a callus (non-embryogenic culture) were collected and transferred to 50-mL Erlenmeyer flasks filled with 40 mL of an E5 medium and closed with rubber stoppers. The air sample was taken for analysis after 24 h according to Kępczyńska et al. (2009). For each combination that was tested, an air sample of 1 mL was injected into a Hewlett Packard 5890 Series II gas chromatograph that was fitted with an FID detector and a stainless steel column (6 fit × 1.8 in. × 2.1 mm). All measurements were taken in triplicate.

### Content of indolic compounds

In order to roughly estimate the IAA content, a colorimetric technique was applied to detect any indolic compounds, including IAA (Bric et al. 1991). Explants of Col-0 and the *erf022* mutant that were induced for 5 days on an E5 medium were analysed. The concentration of indolic compounds was determined by using a calibration curve of pure IAA as the standard following linear regression analysis. Each analysis was carried out in three replicates.

### Gene expression analysis

An RNAqueous Kit (Ambion) was used to isolate total RNA from fresh (0 days) and in vitro cultured explants that were induced on media with different a hormone content to promote alternative developmental pathways. Tissue samples were collected during different culture stages, from 0 to 15 days. Depending on the age of the culture, from 250 IZEs (0 days) to 20 (15 days) IZE-derived cultures were used for RNA isolation in one biological replicate. The concentration and quality of the isolated RNA was evaluated using an ND-1000 NanoDrop spectrophotometer. RNAs were treated with RQ1 RNase-free DNase I (Promega) following the manufacturer’s instructions. First-strand cDNA was produced using a RevertAid First-Strand cDNA Synthesis Kit (Fermentas). The product of the reverse transcription was diluted with water at a 1:1 ratio and 1 µL of this solution was used for RT-PCR reactions. In the real-time RT-qPCR reactions, LightCycler Fast-Start DNA Master SYBR Green I (Roche) was applied in the LightCycler 2.0 (Roche) real-time detection system. The primers that were relevant to the genes being studied were used in the RT-PCR analysis (Table 1).

**Table 1:** Primers used for RT-PCR reactions

Gene	Primer sequence
*ERF022* (At1g33760)	pF ACGAGATTACCGCTTCAACG pR AAGTTGAGATTGGTCCCACG
*ACS7* (At4g26200)	pF TTGGAGAAGAAGAATCCAGAAGG pR AGTTCGTTAGCGGCGGTG
*ERF1* (At3g23240)	pF CCTTCCGATCAAATCCGTAA pR ACCCTCTCATCGAGAAAGCA
*ETR1* (At1g66340)	pF GTCTCCGAGTTGTGTCCCAT pR TGATACGGGTTTGAGCAACA
*LEC2* (At1g28300)	pF AGGGAAAGGAACCACTACGAA pR CAGTGGTGAGGTCCATGAGAT
*YUC1* (At4g32540)	pF CGGAACACCGTTCATGTGT pR CCGGTGACATTTTTCAGCTC
*YUC4* (AT5g11320)	pF AACTCCCGTTCTTGATGTCG pR AAAAACTATTCTCCTTAAGCCAATC
*At4g27090*	pF GTCGTTATCGTCGACGTTGTT pR CCTCGATCAAAGCCTTCTTCT

Relative RNA levels were calculated and normalised to an internal control, the *At4g27090* gene encoded 60S ribosomal protein. The control gene exhibited a constant expression pattern (*C*_T_ = 18 ± 1) in all tissue samples that were analysed. The plant tissues for the real-time RT-qPCR analysis were produced in three biological repetitions and two technical replicates of each repetition were carried out. Relative expression level was calculated using $2^{{ - \Delta \Delta C_{\text{T}} }}$, where ∆∆*C*_T_ represents $\Delta C_{{_{\text{T}} }}^{\text{reference\; condition}} - \Delta C_{{_{\text{T}} }}^{\text{compared\; condition }}$.

### Statistical analysis

ANOVA, Kruskal–Wallis and *U* Mann–Whitney’s statistical test were applied to calculate any significant differences (at *P* = 0.05) between the combinations. The graphs show the averages with the standard deviation; statistical analysis was performed with the medians.

## Results

### *ERF022* expression level and culture capacity for SE

**Fig. 1:**
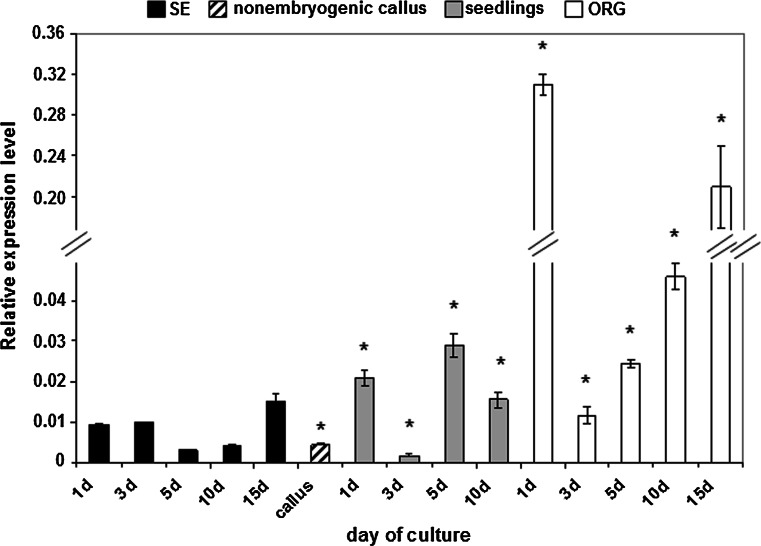
Expression level of the *ERF022* gene in Col-0 culture. IZE explants induced towards somatic embryogenesis (SE), shoot organogenesis (ORG) and seedling development (E0) and the non-embryogenic callus. Relative transcript level was normalised to an internal control (*At4g27090*) and calibrated to 0 days culture. * Value significantly different from the embryogenic culture of the same age (*P* < 0.05; *n* = 3 ± SD)

Our previous study on SE-related TF transcriptome indicated a significantly reduced activity of *ERF022* in Col-0 explants that had been subjected to embryogenic induction (Gliwicka et al. 2013). To confirm that the inhibition of *ERF022* is specific for the induction of SE, the gene expression level was tracked in Col-0 IZE explants that had been cultured in vitro on different media in order to induce alternative morphogenic pathways including SE, shoot ORG and seedling development (Kraut et al. 2011). The results indicated that a down-regulation of *ERF022* transcription could be observed in all of the developmental pathways (SE, ORG and E0) induced. However, the explants induced towards SE displayed the strongest reduction (up to 320-fold) in the gene activity (Fig. 1). Interestingly, the lowest *ERF022* expression was observed in non-embryogenic calluses that were produced occasionally on explants that had failed in the induction of SE. This observation together with the significantly impaired embryogenic capacity of the *erf022* knock-out mutant (Gliwicka et al. 2013) suggest that although a drastic down-regulation of *ERF022* was found to be associated with SE-induction, the expression of *ERF022* at a specific level is required for the efficient induction of SE. In addition, a significantly different from a highly embryogenic Col-0 culture level of *ERF022* expression was indicated in cultures of the *lec2*, *tan2*-*1* and *cbp20* mutants that are significantly impaired in the SE response (Ledwoń and Gaj 2009; Baster et al. 2009) (Supplemental Fig. S1). For these reasons, it can be assumed that a reduced and finely tuned level of *ERF022* activity seems to be required for effective embryogenic induction in a culture of IZE explants.

### *ERF022* and ethylene

**Fig. 2:**
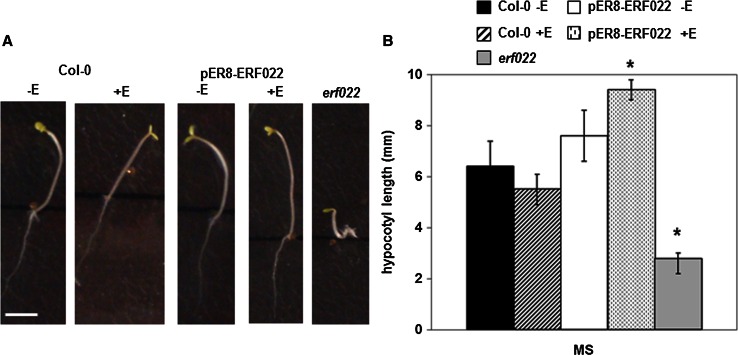
Phenotypes of 3-day-old etiolated seedlings that resulted from different *ERF022* expression levels in Col-0, pER8-ERF022 transgenic line and *erf022* mutant. An elongated, thin stature of a transgenic seedling that overexpressed *ERF022* and a short, thickened stature of an *erf022* mutant seedling (**a**); *ERF022*-modulated hypocotyl length (**b**). *ERF022* overexpression was induced with β-estradiol (+E). * Values significantly different from Col-0 seedlings (*P* < 0.05; *n* = 3 ± SD). *Scale bars* 3 mm

We observed that ACC modulates *ERF022* expression in the seedlings, which confirms the assumed relation of the gene with ethylene (Supplemental Fig. S2). The ethylene-related functions of *ERF022* were further supported by the phenotypic analysis of the seedlings of the *erf022* mutant and the transgenic pER8-ERF022 line that had been grown in the dark. The analysis indicated that the *erf022* seedlings displayed a partial triple response phenotype (Fig. 2). The hypocotyls and roots of the mutant seedlings were distinctly shorter and thicker but the exaggerated apical hook, which is expected in a triple response, was not observed. In contrast to the *erf022* mutant, seedlings that overexpressed *ERF022* developed significantly elongated roots and hypocotyls. The observed changes in the morphology of seedlings that were found to be associated to *ERF022* activity implied a link between the analysed gene and ethylene metabolism and/or signalling. Based on this assumption, the ethylene content was evaluated in etiolated seedlings of the *erf022* mutant and pER8-ERF022 line. The results indicated a significant twofold increase of ethylene in the *erf022* mutant seedlings (Table 2a). All of the analyses of the *erf022* seedlings, including their partial triple response phenotype and elevated ethylene content, imply that *ERF022* negatively controls ethylene production.

**Table 2:** Ethylene content (nmol flask^−1^) in 3-day-old etiolated seedlings with a different level of *ERF022* activity in Col-0, pER8-ERF022 transgenic line and *erf022* mutant (*a*) and in the IZE-derived embryogenic culture and the non-embryogenic callus of Col-0 (*b*)

Tissue type	Col-0		PER8_ERF022		*Erf022*
	−E	+E	−E	+E	
*a*					
3-day-old seedlings	6.6 ± 2.3	6.5 ± 3.5	7.7 ± 1.7	7.1 ± 0.5	14.7* ± 2.9
*b*					
Embryogenic culture	24.4 ± 6.6	na	na	na	na
Non-embryogenic callus	44.0* ± 5.6	na	na	na	na

*ERF022* overexpression was induced with β-estradiol (+E), *na* not analysed

* Values significantly different from Col-0 (a) or the embryogenic culture (b); *P* < 0.05; *n* = 3

### Ethylene and culture capacity for SE

**Fig. 3:**
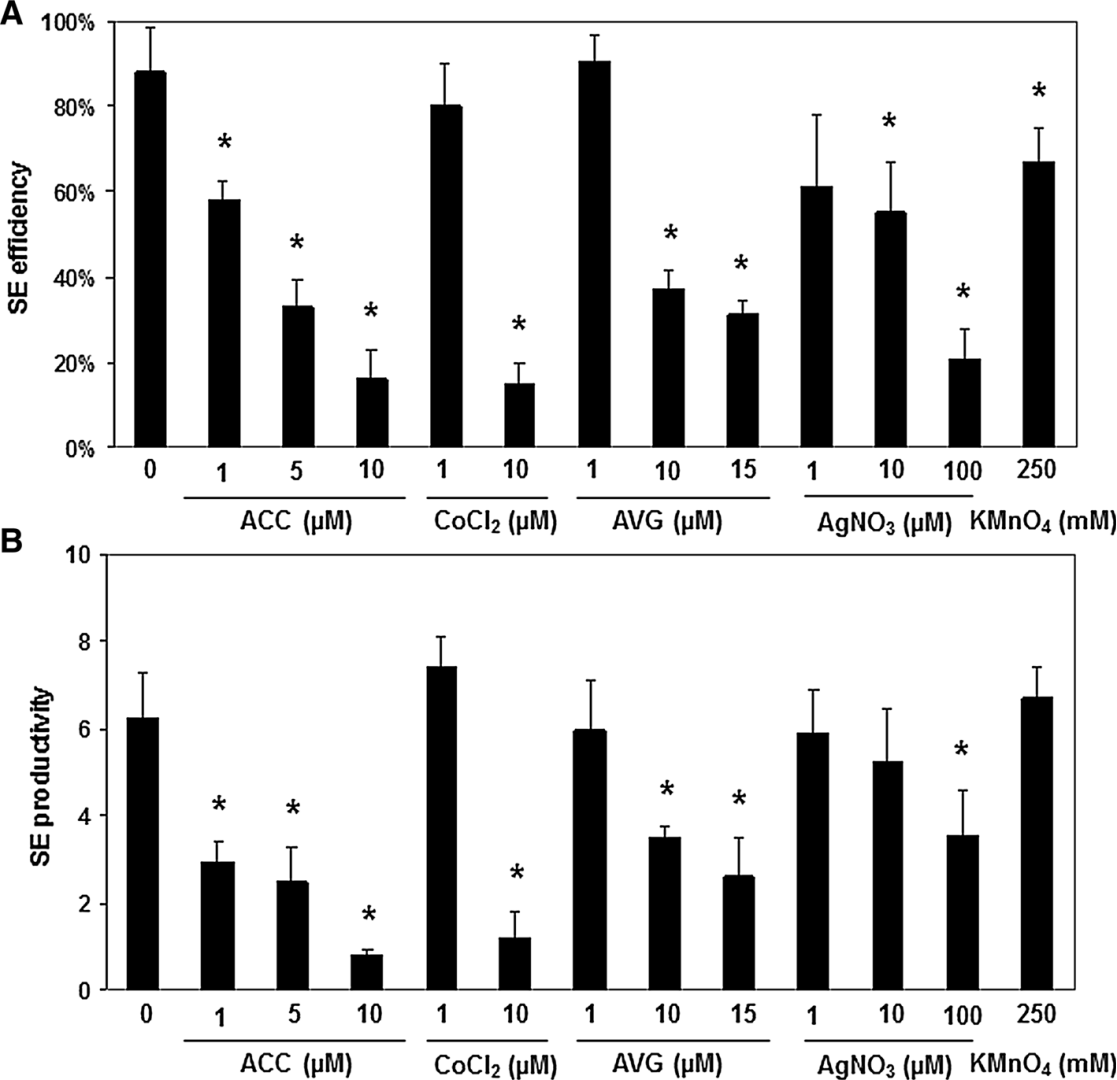
Influence of ethylene modulators ACC, CoCl_2_, AVG, AgNO_3_ and KMnO_4_ on the embryogenic potential of the IZE explants of Col-0 that were induced on an E5 medium. SE efficiency (**a**) and SE productivity (**b**) were evaluated. * Values significantly different from the control (0 μM) culture (*P* < 0.05; *n* = 3 ± SD)

The elevated ethylene content in the *erf022* seedlings that was indicated together with a reduced embryogenic response of this mutant (Gliwicka et al. 2013) suggest that ethylene can negatively interfere with the embryogenic potential of Arabidopsis explants. The assumed inhibitory effect of ethylene seems to be specific to the embryogenic pathway of development as IZE explants of *erf022*, in contrast to SE, retained an efficient shoot ORG (Supplemental Fig. S3). Accordingly, it can be speculated, that in contrast to SE induction, ethylene may not significantly affect shoot regeneration in IZE culture but this hypothesis needs further verification. The negative impact of ethylene on the embryogenic potential of Arabidopsis was further supported by the significantly (almost twofold) elevated ethylene content that was observed in non-embryogenic vs embryogenic culture of the same age (Table 2b). To gain more insight on the relation between ethylene and culture capacity for the induction of SE, the effect of ethylene modulators on the embryogenic potential of WT culture were evaluated. A variety of chemicals that increase (ACC) or decrease (CoCl_2_, AVG) ethylene production and interfere with ethylene perception (AgNO_3_) and activity (KMnO_4_) were applied to the SE-induction medium. It was found that all of the agents significantly reduced the embryogenic response of the WT culture in terms of SE efficiency and productivity (Fig. 3). The observed effects were dependent on the concentration of the ethylene modulators that were applied.

**Fig. 4:**
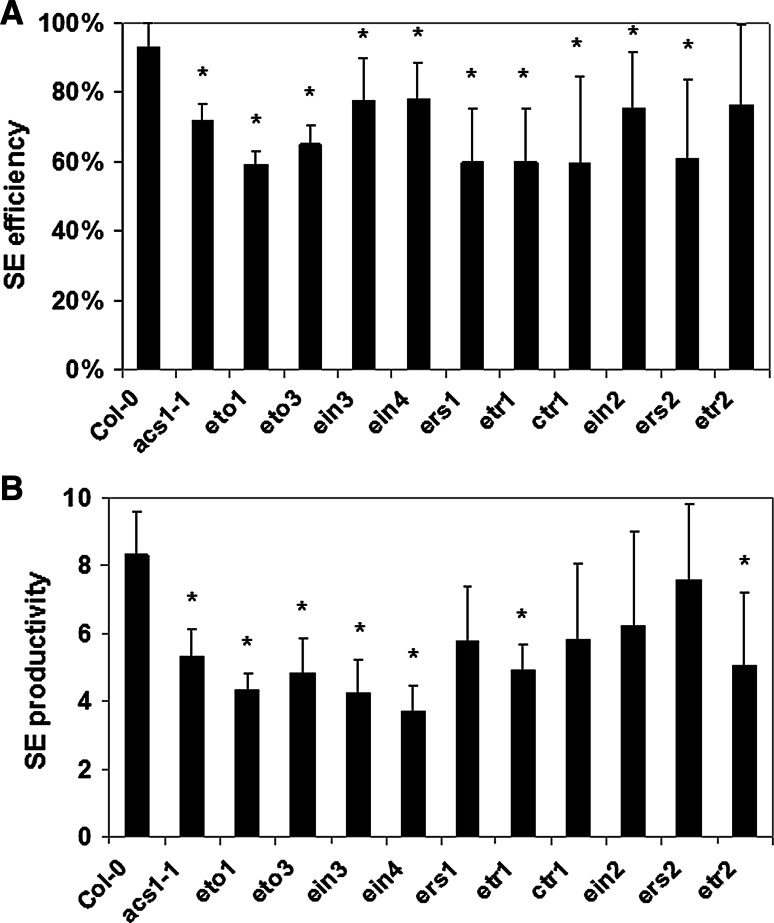
Capacity for SE in the cultures of mutants that were impaired in ethylene biosynthesis (*acs1*-*1*, *eto1*, *eto3*) and perception (*ein4*, *etr1*, *etr2*, *ers1*, *ers2*, *ctr1*, *ein2*, *ein3*) and their parental genotype, Col-0. SE efficiency (**a**) and SE productivity (**b**) of the IZE explants culture that was induced on an E5 medium. * Values significantly different from Col-0 (*P* < 0.05; *n* = 3 ± SD)

To further confirm the impact of ethylene on SE, the mutants in the ethylene biosynthesis, including the *acs1*-*1* with a low ethylene level (Tsuchisaka et al. 2009) as well as *eto1* and *eto3*, which are characterised by increased ethylene production (Woeste et al. 1999) were analysed in vitro in respect to their embryogenic potential. In addition, the SE-capacity was also evaluated in the mutants that were defective in genes encoding ethylene receptors (*EIN4*, *ETR1*, *ETR2*, *ERS1*, *ERS2*) and those that negatively (*CTR1*) or positively (*EIN2* and *EIN3*) control ethylene signalling. The analysis indicated that all of the ethylene-related mutants displayed a significantly reduced SE efficiency and/or SE productivity (Fig. 4).

Taken together, the results on the impact of ethylene modulators and ethylene-related mutations on the embryogenic potential of culture suggest that a properly balanced ethylene production, perception and signalling seem to be required for the efficient embryogenic capacity of Arabidopsis explants.

### ERF022 down-regulates *ACS7* and *ERF1*

**Fig. 5:**
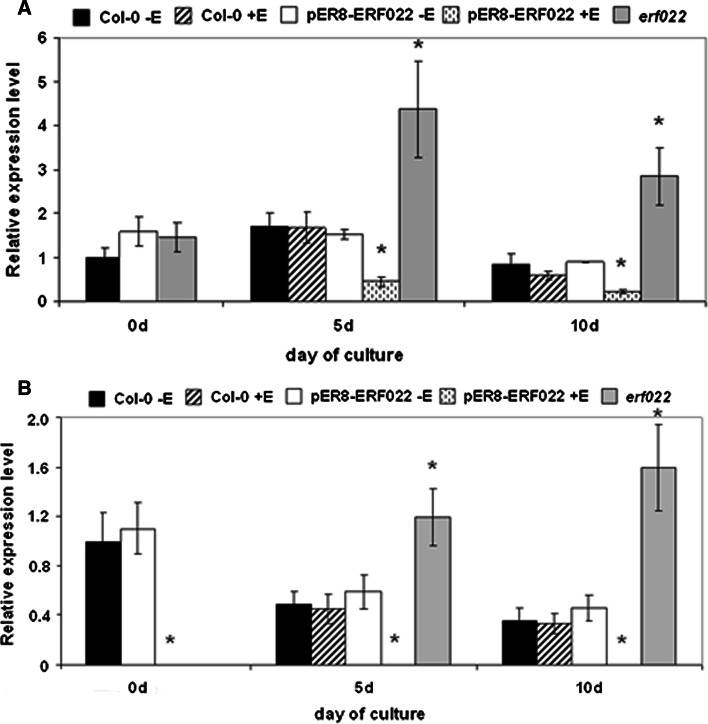
Expression analysis of *ERF022*-related genes. Expression level of *ACS7* (**a**) and *ERF1* (**b**) in the IZE-culture that was induced on an E5 medium in Col-0, pER8-ERF022 and the *erf022* mutant. *ERF022* overexpression was induced with β-estradiol (+E). Relative transcript level was normalised to an internal control (*At4g27090*) and calibrated to 0 days Col-0 culture. * Values significantly different from the Col-0 culture of the same age (*P* < 0.05; *n* = 3 ± SD)

**Fig. 6:**
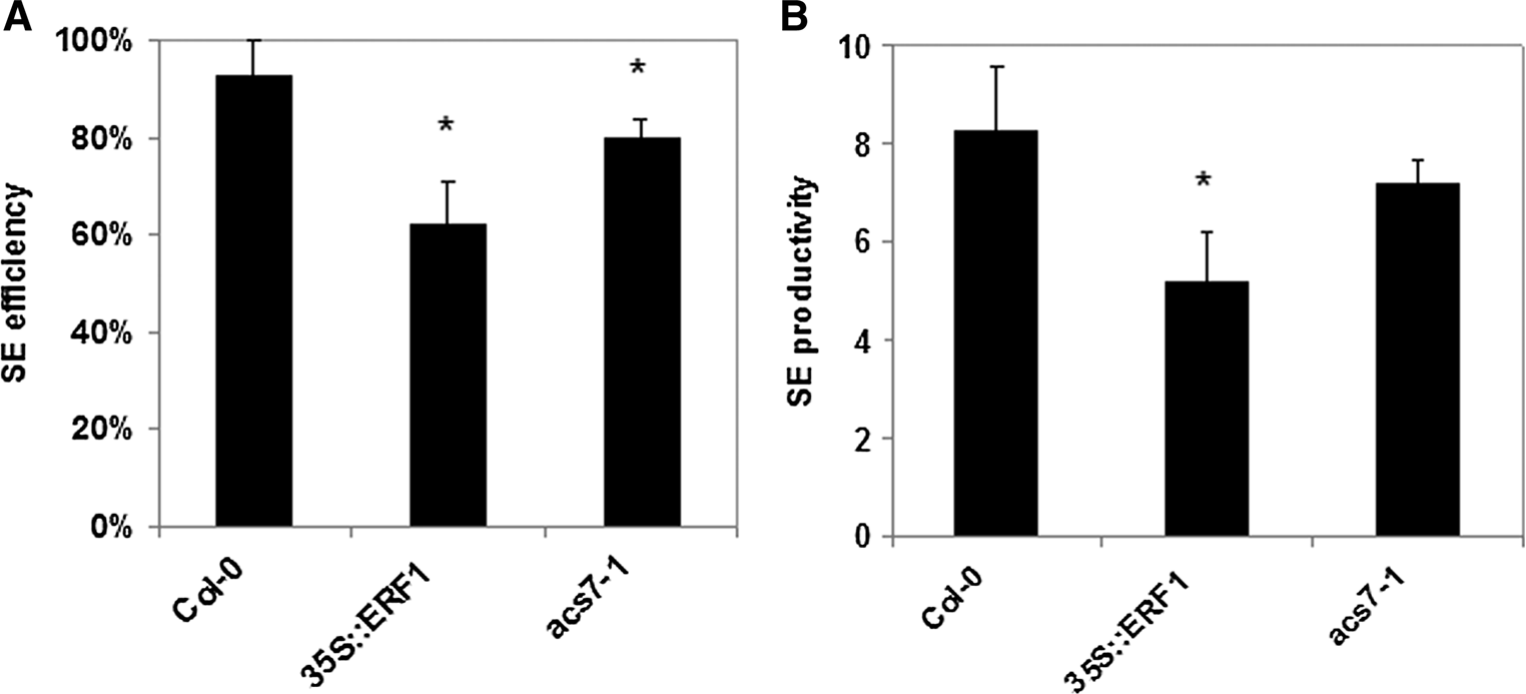
SE-capacity of the *acs7*-*1* mutant, 35S::ERF1 transgenic line and Col-0. SE efficiency (**a**) and SE productivity (**b**) of the IZE explants that were cultured on an E5 medium was evaluated. * Values significantly different from Col-0 (*P* < 0.05; *n* = 3 ± SD)

In order to reveal the *ERF022*-related genetic components, the co-expressed genes were searched for using Expression Angler analysis (http://bar.utoronto.ca/). The analysis indicated four ethylene-related genes, including genes that encode ACC synthases (*ACS8* and *ACS7*), which participate in ethylene biosynthesis and the transcription factors (*ERF1* and *ERF5*) that are involved in ethylene signal transduction. The results of a semi-qRT-PCR analysis of 10-day-old seedlings suggested the inhibition of *ACS7* and *ERF1* gene expression in response to *ERF022* overexpression (Supplemental Fig. S4a). Further analysis using real-time RT-qPCR confirmed the *ERF022*-modulated expression of *ACS7* and *ERF1* in Col-0 embryogenic culture (Fig. 5a, b). It was observed that the *erf022* mutation resulted in a distinct increase in the expression of *ACS7* and the activation of *ERF1* transcription in the cultured explants. In contrast to *erf022*, in the culture that overexpressed *ERF022*, the transcription of *ERF1* was not indicated and a reduction of *ACS7* transcription was observed. To confirm the involvement of *ACS7* and *ERF1* in the induction of SE, we evaluated the embryogenic potential of the *acs7*-*1* insertional mutant and the 35S::ERF1 overexpressor line. The analysis showed that both genotypes have a significantly reduced embryogenic potential (Fig. 6). Taken together, the results imply that the *ERF022* negatively controls the expression of genes involved in ethylene biosynthesis (*ACS7*) and signalling (*ERF1*) and thus, a repressive function of *ERF022* in ethylene-related pathways can be assumed.

### ERF022 up-regulates *ETR1*

**Fig. 7:**
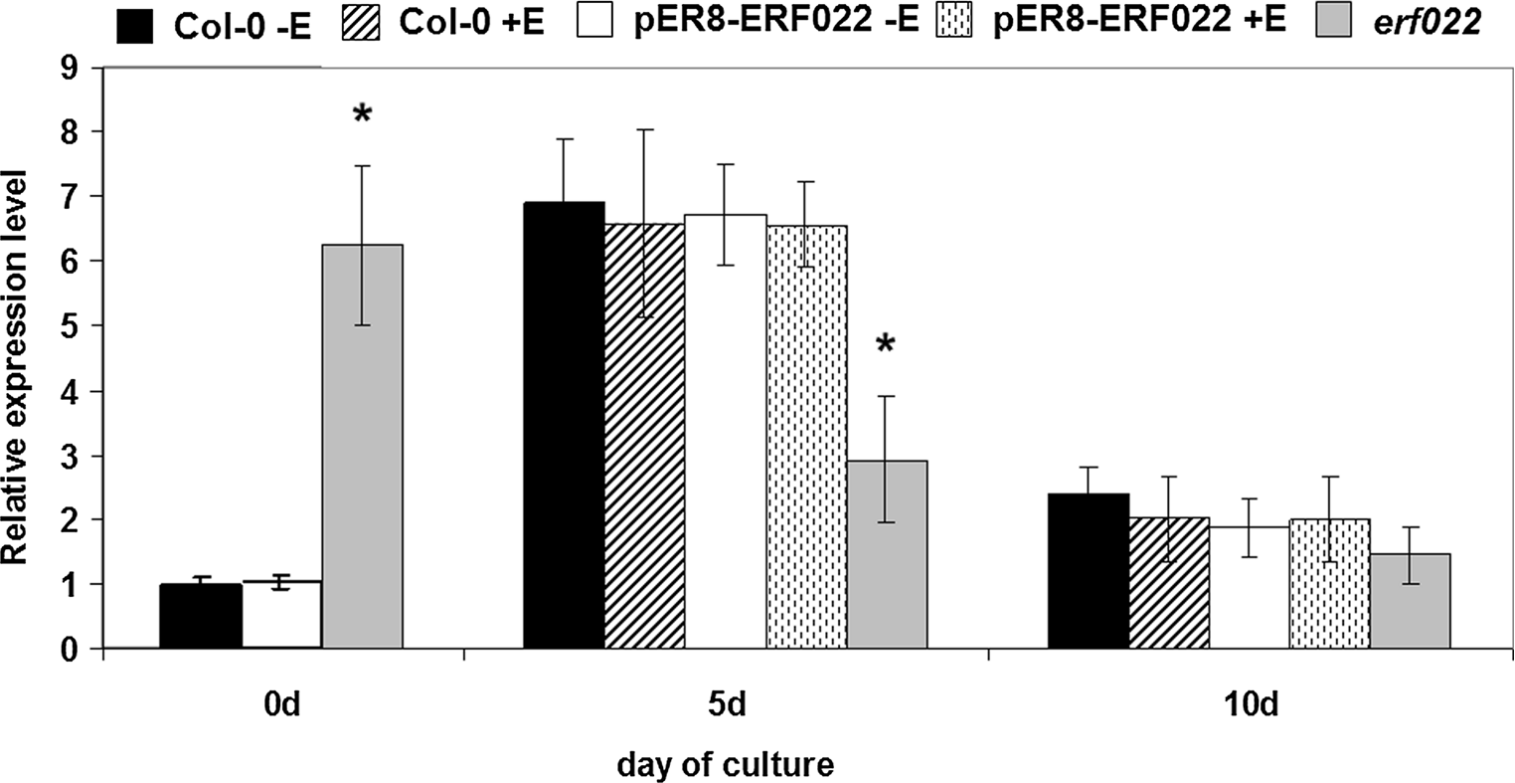
Expression level of the *ETR1* gene in the IZE-culture on an E5 medium of Col-0, pER8-ERF022 transgenic line and the *erf022* mutant. *ERF022* overexpression was induced with β-estradiol (+E). Relative transcript level was normalised to an internal control (*At4g27090*) and calibrated to the 0 days Col-0 culture. * Values significantly different from the Col-0 culture of the same age (*P* < 0.05; *n* = 3 ± SD)

Because of the suggested impact of *ERF022* on ethylene signalling, presumably via down-regulation of *ERF1*, the analysis was extended to other genes that act in the signal transduction pathway, including those that encode ethylene receptors (*EIN4*, *ETR1*, *ETR2*, *ERS1*, *ERS2*), the negative (*CTR1*) and positive (*EIN2*) regulators of ethylene signalling and a direct regulator of *ERF1* (*EIN3*). Expression analysis of these genes showed that the transcription of most of them is not affected in the pER8-ERF022 transgenic line (data not presented) with the exception of *ETR1*, which was up-regulated in response to *ERF022* overexpression (Supplemental Fig. S4b). The positive regulatory impact of *ERF022* on *ETR1* activity was also suggested by the drastically low level of *ETR1* transcripts that were observed in the *erf022* mutant seedlings. However, in contrast to the seedlings, the IZE explants of the *erf022* mutant accumulated higher (more than sixfold higher than Col-0) level of *ETR1* transcripts (Fig. 7). This observation suggests that the regulatory relations between *ETR1* and *ERF022* seem to be tissue/organ specific.

*ETR1* expression was also monitored in the IZE-culture and substantial differences in the gene expression profile were noticed between Col-0 and *erf022* cultures. In contrast to the highly embryogenic Col-0 culture, which displayed a strong (sevenfold) stimulation of *ETR1* transcription, in the *erf022* culture, which was defective in an embryogenic response, a significant (twofold) reduction in the *ETR1* transcript level was observed during early stage (5 days) of SE induction. It was also noticed that, in contrast to the considerable impact of the *erf022* knock-out mutation on *ETR1* activity, the overexpression of *ERF022* did not affect *ETR1* expression.

### *ERF022* and MeJA

**Fig. 8:**
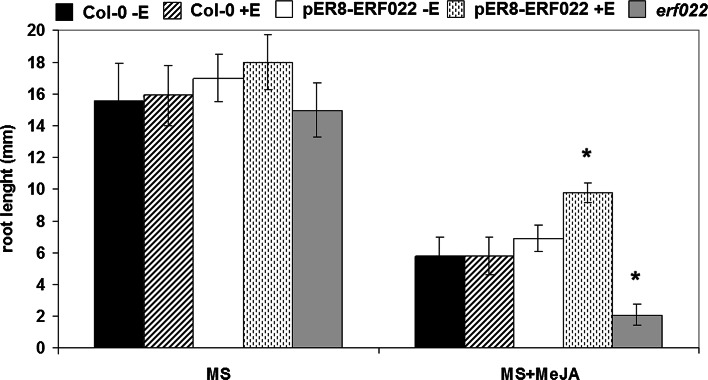
Root length of 7-day-old seedlings of Col-0, pER8-ERF022 and *erf022* in response to MeJA. MS medium was supplemented with 10 µM of MeJA. *ERF022* overexpression was induced with β-estradiol (+E). * Values significantly different from Col-0 (*P* < 0.05; *n* = 3 ± SD)

*ERF1* expression is induced in response to ethylene or jasmonate alone or in combinations (Lorenzo et al. 2003). Because the present results imply a regulatory relation between *ERF022* and *ERF1*, the involvement of *ERF022* in jasmonate signalling cannot be excluded. It is expected that the mutants that are associated with the synthesis or signal transduction of jasmonate will produce a drastically reduced root in response to MeJA treatment (Staswick et al. 1992). Therefore, the root lengths of Col-0, pER8-ERF022 and *erf022* mutant seedlings were estimated under MeJA treatment. The results showed that MeJA treatment significantly inhibited root elongation in the seedlings and this phenotype was considerably influenced by the *ERF022* expression level (Fig. 8; Supplemental Fig. S5). After MeJA treatment, the roots of the *erf022* and pER8-ERF022 seedlings were 2.9-fold shorter and 1.6-fold longer than those of Col-0, respectively. These results suggest that *ERF022*, in addition to the assumed relation with ethylene, may also be involved in the MeJA signal transduction pathway possibly via the regulation of *ERF1*.

### *ERF022* and stress

**Fig. 9:**
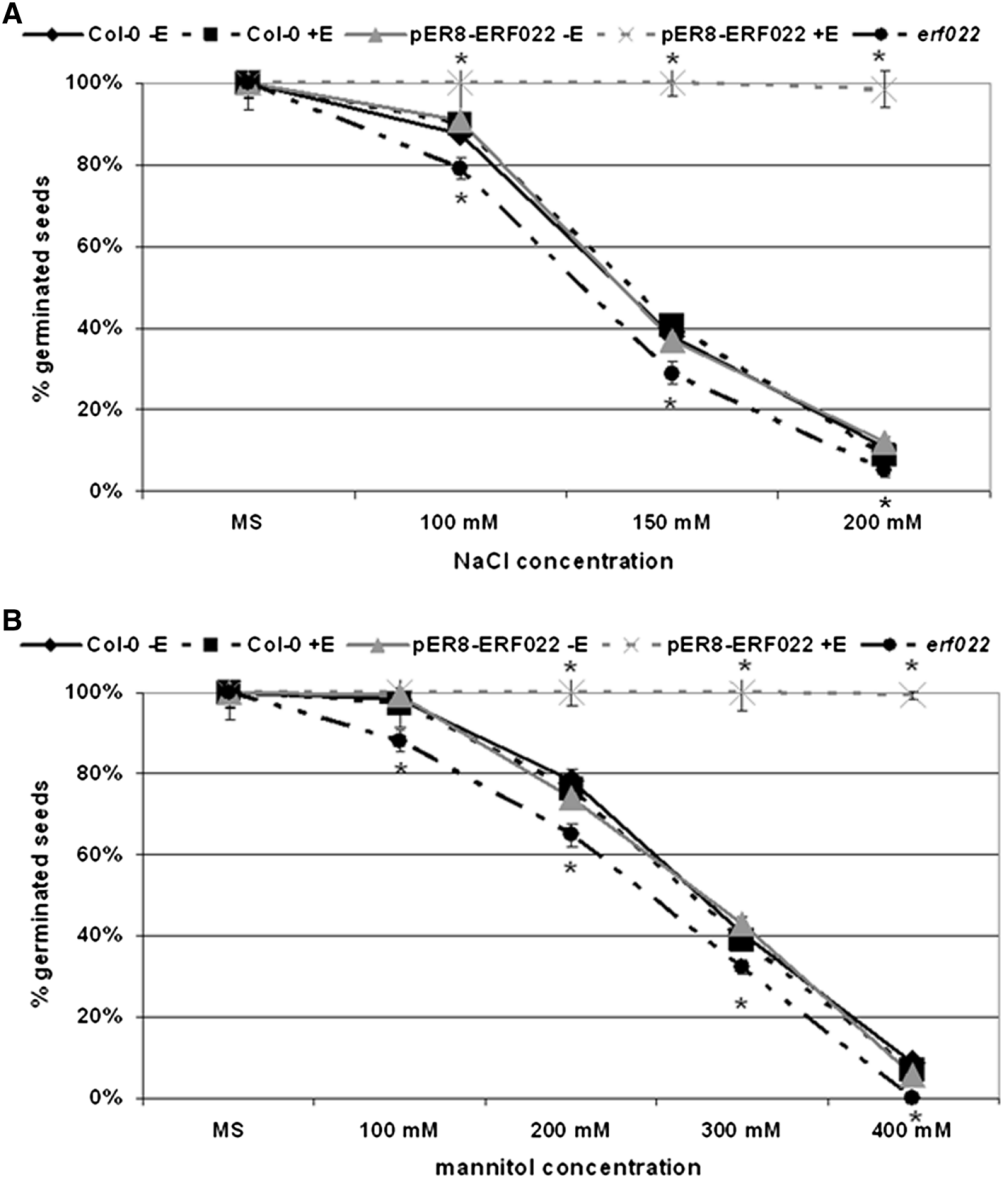
Seed germination frequency of Col-0, pER8-ERF022 transgenic line and the *erf022* mutant under stress that was induced with NaCl (**a**) and mannitol (**b**). *ERF022* overexpression was induced with β-estradiol (+E). * Values significantly different from the Col-0 seedlings (*P* < 0.05; *n* = 3 ± SD)

The results imply a regulatory link between *ERF022* ethylene and jasmonate and *ETR1*, which are involved in plant responses to stress factors (Wang et al. 2008). Therefore, the sensitivity of the seeds to salt and osmotic stress in respect to the *ERF022* expression level was evaluated. In order to evaluate this, the germination ability of Col-0, pER8-ERF022 and *erf022* seeds under various concentrations of NaCl and mannitol was estimated. The analysis clearly demonstrated a positive relation between the *ERF022* activity and the tolerance of the seeds to the salt and osmotic stresses (Fig. 9). Seeds that overexpressed *ERF022* were able to germinate with 100 % frequency in all of the stress-induced combinations, while the tolerance of seeds to NaCl and mannitol was substantially reduced in *erf022*. The results of this experiment clearly indicate that the *ERF022* expression level significantly affects the tolerance of seeds to abiotic stress and thus suggests the involvement of *ERF022* in the mechanisms that are associated with the plant responses to stress.

### *ERF022* relation with *LEC2*

**Fig. 10:**
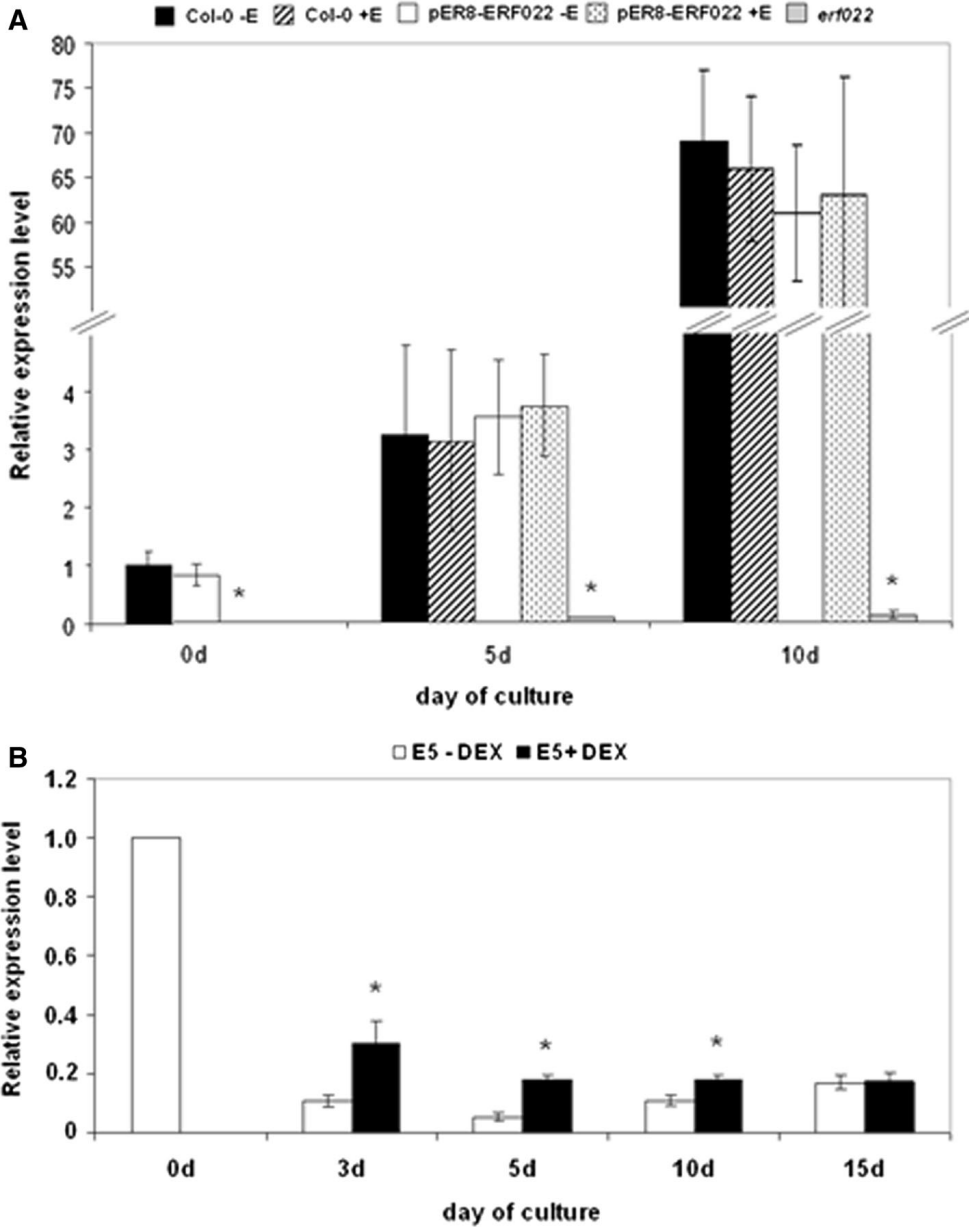
The regulatory relation between *ERF022* and *LEC2*. Expression level of the *LEC2* gene in the IZE-culture of Col-0, pER8-ERF022 transgenic line and the *erf022* mutant that was induced on an E5 medium (**a**). *ERF022* overexpression was induced with β-estradiol (+E). Expression level of *ERF022* in the transgenic 35S::LEC2-GR culture that was induced an on E5 medium (**b**). *LEC2* overexpression was induced with dexamethasone (+DEX). Relative transcript level was normalised to an internal control (*At4g27090*) and calibrated to the 0 days of Col-0 (**a**) or 0 days of 35S::LEC2-GR (**b**). * Values significantly different from control culture at the same age (*P* < 0.05; *n* = 3 ± SD)

Considering the reduced morphogenic potential of the *erf022* explants that was specific to the induction of SE, the relation between *ERF022* and *LEC2*, a key regulator of embryogenic transition in somatic cells of Arabidopsis was explored (Ledwoń and Gaj 2009). The expression level of *LEC2* was evaluated in embryogenic cultures with different levels of activity of the *ERF022* gene. The analysis indicated that *LEC2* expression in the *erf022* explants and the derived culture was strongly inhibited (up to 500-fold) (Fig. 10a). In contrast to the *erf022* mutation, the *ERF022* overexpression did not change the transcript level of the *LEC2* gene. To further explore the regulatory relation between these genes, the *ERF022* transcript level in the culture that overexpressed *LEC2* was evaluated and a significant stimulation of the *ERF022* expression level was observed in the transgenic 35S::LEC2-GR culture treated with DEX (Fig. 10b). Taken together, the results imply the existence of regulatory interactions between *ERF022* and *LEC2*. The activity of *ERF022* seems to be required for *LEC2* expression and *LEC2* transcripts are supposed to positively control *ERF022* transcript level. Because *ERF022* seems to negatively control ethylene biosynthesis, the impact of ACC on *LEC2* expression in Col-0 explant culture was analysed. The analysis indicated a strong reduction of the *LEC2* transcription in the cultured explants in response to ACC treatment (Supplemental Fig. S6). Therefore, it appears that ethylene negatively controls *LEC2* expression in the embryogenic culture and that *ERF022* may be involved in this regulatory relation.

### *ERF022* and auxin

**Fig. 11:**
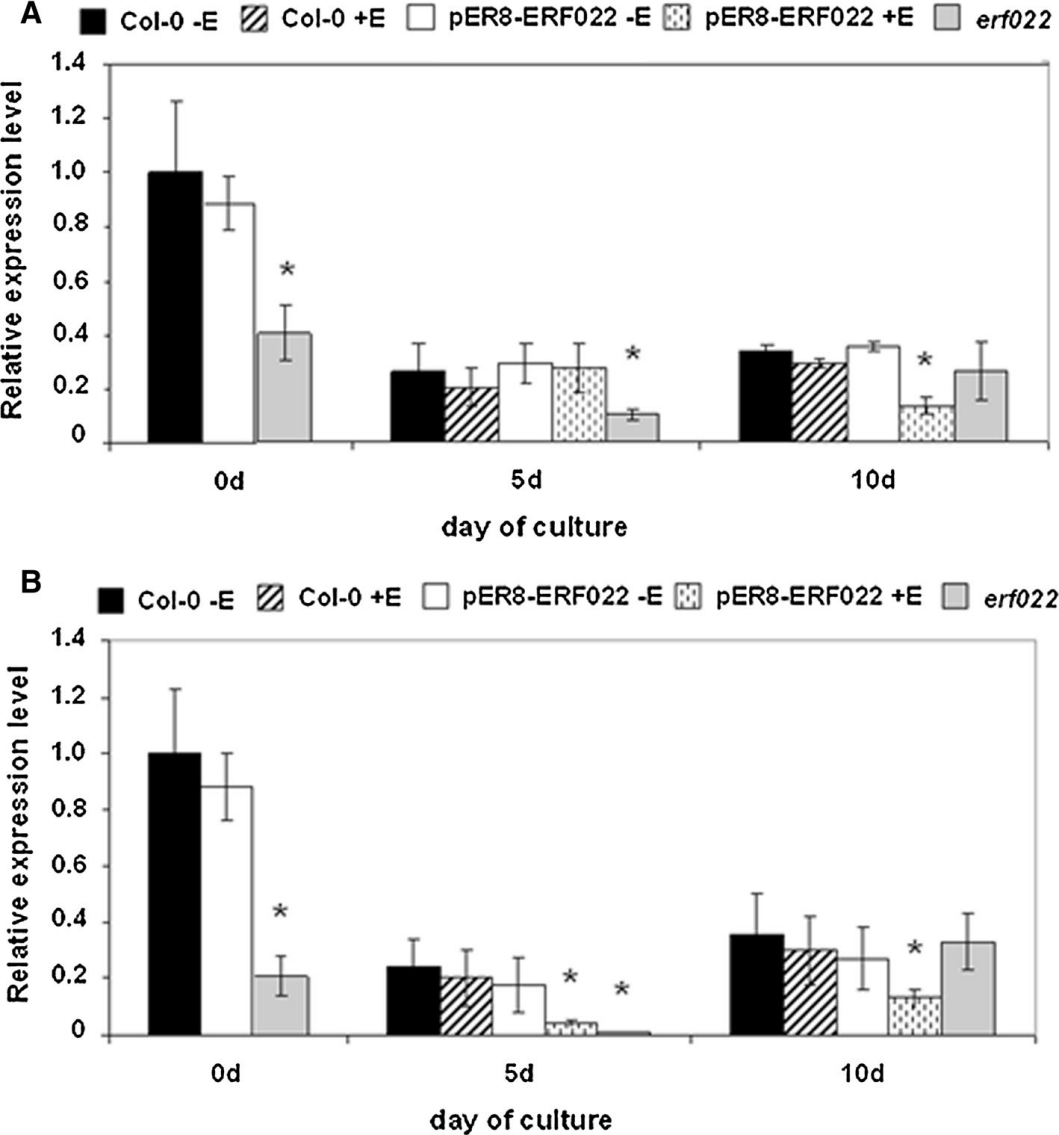
Expression level of the *YUC1* (**a**) and *YUC4* (**b**) genes in the IZE-derived culture of Col-0, pER8-ERF022 transgenic line and the *erf022* mutant that was induced on an E5 medium. *ERF022* overexpression was induced with β-estradiol (+E). Relative transcript level was normalised to an internal control (*At4g27090*) and calibrated to the 0 days Col-0 culture. * Values significantly different from Col-0 at the same age (*P* < 0.05; *n* = 3 ± SD)

A relation between *ERF022* and auxin can be expected since in the embryogenic culture of Arabidopsis the *LEC2* gene, which was found to be presumably under the control of *ERF022*, regulates the induction of SE via the stimulation of the auxin biosynthesis *YUCCA* (*YUC*) genes (Wójcikowska et al. 2013). To reveal any further components of the *ERF022*-related mechanism of the induction of SE, an attempt was made to find a link between this gene and auxin biosynthesis and *YUC* genes (*YUC1*, *YUC4* and *YUC10*) of the *LEC2*-stimulated activity in SE (Wójcikowska et al. 2013) were analysed in respect to the *ERF022* expression level. The analysis showed that the knock-out mutation in *ERF022* leads to a reduced expression in two of the *YUC* genes that were analysed, *YUC1* and *YUC4* (Fig. 11), which suggests a decreased auxin level in the *erf022* mutant. To verify this hypothesis, the level of indolic compounds was estimated in the *erf022* mutant explants cultured on an E5 medium for 5 days. A significantly lower (over 20 %) level of indolic compounds was detected, which infers a decreased IAA level in the *erf022* culture (Table 3). In conclusion, the impact of *ERF022* on the auxin level, presumably via the positive regulation of *LEC2*, which is involved in the control of the SE-involved *YUC* genes, may be suggested. Further analyses are needed to verify this hypothesis.

**Table 3:** Endogenous level of indolic compounds (µg/g of fresh tissue) in the IZE-culture of Col-0 and the *erf022* mutant

	Col-0	*erf022*
IZE-derived culture (5 days)	218.67 ± 19.73	173.18 ± 20.73*

* Values significantly different from Col-0 (*P* < 0.05; *n* = 3)

## Discussion

### Ethylene-related functions of *ERF022*

The *ERF022* expression, which was strongly inhibited in the IZE explants cultured in vitro coupled with the significantly impaired capacity for SE found in the *erf022* mutant culture (Gliwicka et al. 2013), suggests a significant role of *ERF022* in the control of embryogenic transition in somatic cells of Arabidopsis. Therefore, the present study was undertaken in order to verify this hypothesis and to gain insight into the molecular function of the *ERF022* gene.

The high number of ethylene-responsive genes indicated within *ERF* family suggested that the *ERF022* function was related to ethylene (Ohme-Takagi and Shinshi 1995). The results of this study, which include: (i) an increased ethylene content in the *erf022* mutant seedlings; (ii) stimulation of *ERF022* expression in the ACC-treated seedlings; (iii) ethylene-related phenotypes of etiolated seedlings with contrasting *ERF022* activity (*erf022* and pER8-ERF022), support this hypothesis. The significantly increased ethylene level that was detected in the *erf022* mutant suggested that *ERF022* negatively controls ethylene accumulation. The involvement of *ERF022* in ethylene biosynthesis and perception/signalling pathways was considered because of the fact that the mutations in the genes that control all of these ethylene-related pathways were reported to accumulate ethylene in etiolated seedlings (Guzman and Ecker 1990; Woeste et al. 1999).

The analysis of seedlings that overexpressed *ERF022* indicated that two potential down-regulated targets of *ERF022* were involved in ethylene biosynthesis (*ACS7*) and signalling (*ERF1*). *ACS7* is one of 12 *ACS* genes that were identified in the Arabidopsis genome and three of them are non-functional (Tsuchisaka and Theologis 2004). Each *ACS* gene was indicated to have a unique expression profile during plant growth and development and distinct subsets of *ACS* genes are active in response to various developmental, environmental and hormonal factors (Wang et al. 2005). The presence of the DRE motif of the ERF-responding function in the promoter of *ACS7* strengthens the possibility for the regulation of this gene by ERF(s) as was documented for other *ACS* genes in different plants including Arabidopsis (Zhang et al. 2009; Li et al. 2011). For example, ERF11 was indicated to control ABA-modulated ethylene biosynthesis during in vivo plant development through the repression of *ACS2*/*ACS5* genes (Li et al. 2011). However, a repressive domain was not identified in ERF022 thus implying an indirect modulation of *ACS7* activity by this protein. Further components of these interactions, which directly control *ACS7*, remain to be identified among 23 ERFs of a repressive function (Licausi et al. 2013).

A significant and negative impact of *ERF022* activity on the *ACS7* expression level that was observed in the SE-induced explants implies that the auxin treatment is involved in this interaction. Auxin, which is a known inducer of ethylene production, regulates members of the *ACS* multigene family in a tissue-specific manner (Tsuchisaka and Theologis 2004). Recently, *ACS7* was assumed to be engaged in a crosstalk between auxin and ethylene in the root gravity response (Huang et al. 2013). The present results suggest that *ACS7* seems to operate during auxin-induced SE and in support, *acs7*-*1* mutation was found to significantly impair the embryogenic response of the IZE explants.

The results suggest that in addition to ethylene biosynthesis, ethylene signal transduction may also be negatively controlled by *ERF022* via the *ERF1*. *ERF1* regulates expression of a large number of genes responsive to both ethylene and jasmonate and together with other ethylene-related genes it is post-embryonically repressed by *FUS3* in order to delay the vegetative phase transition (Lorenzo et al. 2003; Lumba et al. 2012). A lack of both, the GCC and DRE regulatory motifs in the *ERF1* promoter and a repressive domain in the ERF022 (TAIR—http://www.arabidopsis.org) excludes a direct relation between *ERF022* and *ERF1* and further experiments are needed to identify the mediating genetic elements. *ERF1* transcripts, not detected in the IZEs of *erf022*, were found to be substantially accumulated during the induction of SE, which suggests that in vitro conditions, possibly auxin treatment, affect the activity of *ERF1.* Some observations imply a link between *ERF1* and auxin in plant growth including the ERF1-mediated down-regulation of some *ARF* genes, key regulators of auxin signalling (Lorenzo et al. 2003).

The results suggest that in addition to its indirect repressive function that was postulated for *ERF1* and *ACS7*, *ERF022* may also activate ethylene-related genes. In this study, we found the *ETR1* gene to be up-regulated in the seedlings that overexpressed *ERF022*. *ETR1* encodes one of five ethylene-blocked receptors (ETR1, ETR2, ERS1, ERS2, EIN4) that are associated with the endoplasmic reticulum (Chen et al. 2005). The suggested regulatory relation of *ETR1* and *ERF022* implies that the triple response phenotype of the *erf022* mutant seedlings may result not only from the activation of ethylene synthesis genes, but also from the reduced activity of ethylene receptors, which would cause an altered repressiveness to ethylene and an exaggerated response (Cancel and Larsen 2002). Similar to the *erf022* seedlings that displayed an inhibited *ETR1* expression, an increased ethylene level was also indicated in the seedlings of the ethylene-insensitive *etr1* (ethylene resistant) mutant (Guzman and Ecker 1990). Thus, a feedback inhibition of ethylene biosynthesis by ethylene receptors and posttranscriptional events were proposed to play an important role in regulating ethylene biosynthesis (Woeste et al. 1999).

In contrast to the repressive function of ERFs, the activation of target gene transcription by these TFs has been reported much less frequently, possibly due to the lower specificity and greater diversity of the activating domains (Chen et al. 2005). Interestingly, the *ERF022*-related activation of *ETR1* indicated in seedlings was not observed in the IZE explants that were induced on an E5 medium, which suggests that auxin, tissue type and the developmental program may modify the interaction between *ERF022* and *ETR1*. It was reported that the activity and target specificity of ERF transcription factors strongly depends on the interaction partners that are related to developmental programs and to stress responses as well (Chen et al. 2005). Moreover, tissue- and stress-modulated expression profiles of *ERFs* suggest that the encoded TFs play essential but distinct roles in various plant tissues in response to different environmental cues and developmental contexts (Wang et al. 2014). For example, AtERF73/HRE1 acts as a positive or negative regulator of its target genes in normal and low oxygen conditions, respectively (Yang et al. 2011).

The candidate targets of *ERF022* presented here (*ACS7*, *ERF1* and *ETR1*) imply the gene involvement of in the control of biosynthesis and signalling of ethylene. Such dual functions were reported for some other hormone-related TFs including, *LEC* genes, which are master regulators of zygotic embryogenesis (Harada 2001). *LEC1* and *LEC2* are involved in regulating biosynthesis and signalling of auxin while *FUS3* is indicated to regulate ethylene biosynthesis and signalling via the control of *ACS2* and *ETR1*, respectively (Jia et al. 2014).

Interactions of ERF022 with other proteins are expected due to the rather indirect relation between the gene and the candidate targets. Similarly, a partial triple response phenotype of etiolated *erf022* seedlings enhances this expectation. A partial ethylene response may reflect a lack of interacting ethylene-related protein(s), which are needed to produce the exaggerated curvature of the apical hook. A similar phenotype was observed in seedlings that overexpressed *ERF1* in which the *HOOKLESS1* expression that is required to mediate a full triple response phenotype was absent (Solano et al. 1998).

Moreover, the different binding site motifs identified in a promoter region of *ERF022* (AGRIS; Athena) suggest that the gene can possibly be regulated by a variety of TFs from different families, including WRKY, MYB, ABI3/VP1, LFY, B3 and ARF.

**Fig. 12:**
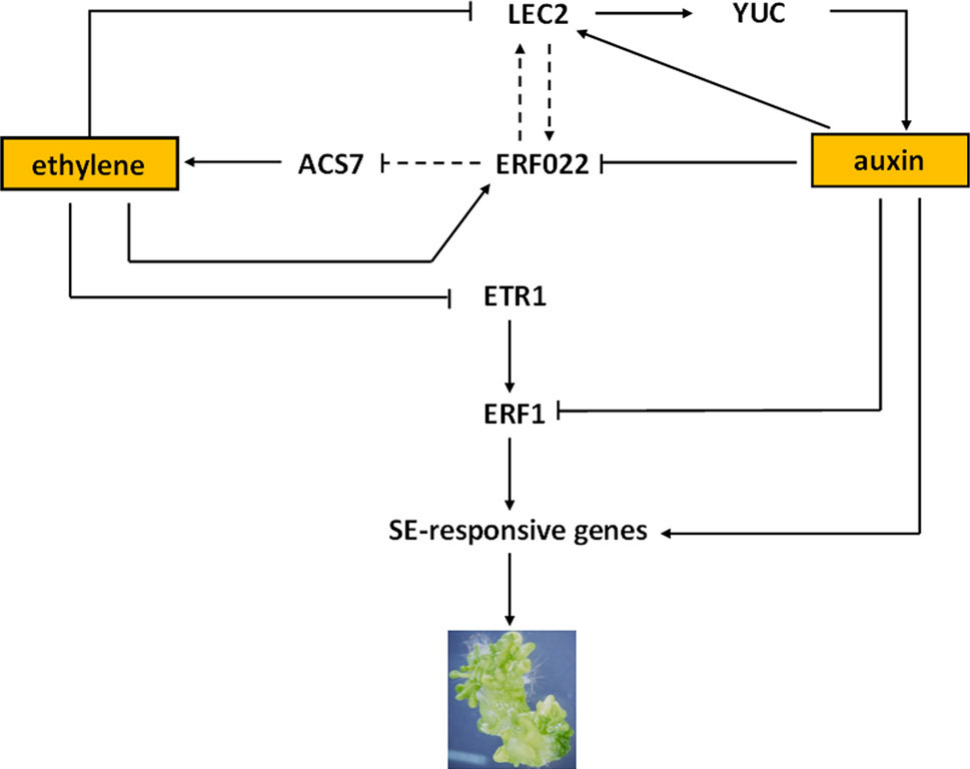
A proposed model of the auxin–ethylene interactions that are controlled by ERF022 and LEC2 and that are involved in the induction of SE. *LEC2* expression is auxin stimulated (Ledwoń and Gaj 2009) and repressed by ethylene (present results, eFP browser). Conversely, *ERF022* expression is repressed by auxin and stimulated by ethylene (present results). *LEC2* activates the *YUCCA* (*YUC*) genes that are involved in auxin biosynthesis (Wójcikowska et al. 2013). *ERF022* may enhance the auxin level through a positive regulatory interaction with *LEC2* (present results). *ERF022* may control ethylene signalling genes, *ETR1* and *ERF1* via the indirect inhibition of ethylene biosynthesis (*ACS7*). An ERF022-mediated decrease in ethylene production may result in the increased activity of *ETR1* and inhibition of *ERF1*, which is consistent with the regulatory relations that were expected between ethylene, ETR1 receptor and ERF1 (Chen et al. 2005). *Black lines* with *arrows* indicate activation and *black lines* ending with *bars* indicate repression. Inferred functions with less experimental evidence are indicated by *dashed lines*

In summary, one of the possible scenarios of the regulatory interactions between *ERF022* and the candidate targets is that ERF022 may impact the ethylene content by having an indirect and negative impact on *ACS7*, which is involved in ethylene biosynthesis. In turn, the ERF022-mediated decrease in ethylene content is expected to stimulate the *ETR1* activity, which results in the down-regulation of *ERF1* (Fig. 12). Further analyses are needed to decipher what other genetic elements interact with *ERF022* in the ethylene-related pathways.

### *ERF022* and stress response

ERF022 was classified to the DREB-like proteins of stress-related functions (Nakano et al. 2006) and we demonstrated that the overexpression of *ERF022* resulted in an enhanced tolerance of seeds to salt and osmotic stress while *erf022* mutant seeds were more sensitive to these stresses. Like many *ERF* genes that are up-regulated in response to abiotic stresses (Saleh and Pages 2003), a considerable induction of *ERF022* expression was observed in NaCl-treated seedlings (KN, MDG—data not presented), which implies a positive relation between *ERF022* activity and the tolerance of seedlings to abiotic stresses. In support of the indicated stress-related function of *ERF022*, its candidate indirect target, *ERF1*, was reported to positively control stress tolerance (Cheng et al. 2013). In addition to *ERF1*, also *ACS7* assumed to be negatively controlled by *ERF022*, may contribute to the *ERF022*-mediated seedling tolerance to stresses because an increased tolerance to salt and osmotic stress was provided by a knock-out mutation in *ACS7* gene (Dong et al. 2011). In the control of plant responses to stress, the *ERF* genes were indicated to integrate a crosstalk between the ethylene and jasmonate-related pathways (McGrath et al. 2005). Consequently, *erf022* seedlings with an elevated ethylene level were indicated to display an enhanced sensitivity to MeJA treatment.

To summarise, the candidate *ERF022* targets *ERF1* and *ACS7* may be involved in the stress-related functions of *ERF022*. Future analyses are needed to confirm the assumed gene interactions and to reveal other genetic components of the ethylene and JA-related pathways that are responsible for the stress tolerance of seedlings controlled by *ERF022*.

### Ethylene and SE

In the present study, SE was induced in a culture of IZE explants treated with 2,4-D and somatic embryos were produced rapidly and efficiently in the presence of auxin and developed mostly without an intervening callus phase (Gaj 2001). We found that the ethylene content was significantly increased in the non-embryogenic calluses that were sporadically produced by explants that had failed in the induction of SE. This result together with the impaired embryogenic response of the mutants with an increased ethylene content (*eto1*, *eto3* and *erf022*) suggests a negative relation between this hormone and the embryogenic potential of the IZE culture. However, treating a culture with chemical modulators (AVG, CoCl_2_), which was expected to reduce the ethylene content, did not significantly improve the embryogenic response of the *erf022* mutant culture (KN and MDG—data not presented). In addition, we also found that other modulators of ethylene (ACC, AgNO_3_ and KMnO_4_) significantly disturbed the embryogenic response of the WT and *erf022* explants. Therefore, it may be reasoned that finely tuned ethylene activity is required for the high embryogenic response in the Arabidopsis IZE explant culture. The requirement of a specific ethylene level and perception for the efficient induction of SE is further supported by the significantly impaired embryogenic potential of numerous ethylene-related mutants that were defective in ethylene production (*eto1*, *eto3*, *acs1*), perception and signal transduction (*ein3*, *ein4*, *ers1*, *etr1*, *ctr1*, *ein2*, *ers2*, *etr2*). Similarly, an analysis of the impact of ethylene on the induction of SE in *Picea mariana* indicated that a decrease in ethylene synthesis may result in adverse effects depending on the embryogenic capacity of the culture. A treatment to decrease the ethylene content was found to improve the embryogenic response in the *P. mariana* lines that had a poor induction of SE, but it impaired SE efficiency in a highly embryogenic culture that displayed a low ethylene content (El Meskaoui and Trembley 2001). Similar to the present results, a negative impact of ACC and mutations that enhance ethylene production (*eto1*) or signalling (*ctr1*) on the induction of SE was also observed in the IZE-derived callus culture of Arabidopsis that produced somatic embryos on an auxin-free medium (Bai et al. 2013). In contrast, a positive effect of ACC on the induction of SE was found in Arabidopsis culture that had been induced from seedlings (Zheng et al. 2013). Differences in ethylene content between the explants (IZEs vs. seedlings) may be responsible for the adverse requirements for ethylene in Arabidopsis cultures that were observed. A significantly lower level of ethylene indicated in the Arabidopsis seedlings than in the siliques with IZEs (Guzman and Ecker 1990) may account for the drastically different embryogenic potential and possibly the different hormone-related genetic programs that are involved in the induction of SE in these explants. In an IZE-culture, SE was induced rapidly and directly from cotyledons (Gaj 2001), while in a much less efficient seedling-derived culture, somatic embryos were induced indirectly from a callus that had formed at SAM (Zheng et al. 2013).

Auxin and ethylene may act synergistically and antagonistically in plant development and the complicated and diverse interactions between these hormones control different developmental processes (Muday et al. 2012). Thus, in addition to the explant type, the mode of auxin treatment that is applied for the induction of SE is expected to drastically modify the ethylene-related mechanisms that are associated with the embryogenic response. Consequently, in contrast to our results, the mutations that disturbed ethylene perception (*etr1*-*3*, *ein2*-*1*) and biosynthesis (*acs2*-*1 acs6*-*1*) did not affect the efficiency of SE in an IZE-derived callus in which somatic embryos were induced in response to the depletion of auxin from a medium (Bai et al. 2013). In light of these observations, it can be assumed that the ethylene-related responses that are involved in the induction of SE may differ among the embryogenic systems that are applied and may depend on the explant type, mode of the induction of SE (direct or indirect) and the auxin regime (a constant presence or withdrawal to induce SE).

### *ERF022* function in SE

The present results ascertain that the *ERF022* seems to be involved in the ethylene-related mechanism of the induction of SE in Arabidopsis. In contrast to many *ERF* genes with a stimulated expression under in vitro culture conditions, including an embryogenic culture of Arabidopsis (Piyatrakul et al. 2012; Gliwicka et al. 2013), *ERF022* was found to be significantly down-regulated in the IZE explants that were induced in vitro towards different morphogenic pathways. This repression of *ERF022* transcription, which is rather unique among *ERFs*, was found especially drastic in the SE-induced IZE and it seems to result from the auxin treatment rather than from abiotic stress per se that is imposed in vitro as a significant up-regulation of *ERF022* was observed in the NaCl-treated seedlings (KN and MDG—data not presented). These considerably contrasting *ERF022* expression profiles imply differences in the ERF022-related genetic mechanisms that operate during auxin-induced embryogenic induction and in the response of plants to abiotic stresses (e.g. NaCl).

With respect to the regulatory impact of *ERF022* on SE, we found that the *erf022* mutation significantly impaired a culture’s capacity for SE (Gliwicka et al. 2013) and a significantly increased ethylene content that is indicated in the mutant may account for the SE-defective phenotype. Interestingly, the overexpression of *ERF022* did not affect the capacity for SE of the explants (Gliwicka et al. 2013) and the ethylene content was not modified in the pER8-ERF022 transgenic plants (present results). It is believed that an overexpression of a TF often fails to induce an informative phenotype, which suggests that a single TF might be insufficient to activate the expression of target genes (Mitsuda and Ohme-Takagi 2009).

Importantly for the genetic mechanism of the induction of SE, the present results suggest that the *ERF022* function may possibly be related to *LEC2*, a positive regulator of SE in Arabidopsis (Gaj et al. 2005; Ledwoń and Gaj 2009). A significant inhibition of the *LEC2* activity was found in the *erf022* mutant with an impaired SE response while the overexpression of *ERF022* did not affect *LEC2* expression. A drastic down-regulation of *LEC2* was observed in the ACC-treated IZE culture (the present results) and seedlings (http://bar.utoronto.ca/efp/cgi-bin/efpWeb.cgi). Therefore, we assume that ethylene negatively controls *LEC2* expression possibly via the *ERF022*-mediated pathway. Although, further experiments are needed to reveal the mechanism of ERF022-LEC2 interactions, both an indirect and a direct mode of regulation can be considered. *ERF022* may activate *LEC2* expression indirectly via the negative regulation of the *ACS7* gene that is involved in ethylene biosynthesis as is suggested here. Importantly, a GCC-box found in an *LEC2* promoter (ATHENA, AGRIS) implies the possibility of the direct regulation of *LEC2* by ethylene-related ERF TFs. Moreover, in a promoter of *ERF022* a RY motif, a seed maturation regulatory sequence targeted by TFs with B3 domain, e.g. LEC2 (Braybrook et al. 2006), was identified. Thus, the possibility of regulatory feedback between *LEC2* and *ERF022* may be expected. We demonstrated that the overexpression of *LEC2* in the IZE embryogenic culture resulted in an enhanced *ERF022* transcription.

Recently, an auxin-related mechanism of the *LEC2* function in SE was described and the gene was found to increase the endogenous auxin level, possibly through the stimulation of the *YUCCA* (*YUC1*, *4* and *10*) genes that are involved in the tryptophan-dependent pathway of auxin biosynthesis (Wójcikowska et al. 2013). In line with the postulated *ERF022*-*LEC2* regulatory relation, the *erf022* mutation was found to result in the reduced activity of *YUC1* and *YUC4*, which was accompanied by a decrease in indolic compounds, including the IAA, level. Thus, a high ethylene level coupled with a low auxin level seems to be responsible for the distinctly decreased embryogenic capacity of *erf022*. Importantly, we found that in contrast to the *erf022* mutant, the level of indolic compounds did not change in the culture that overexpressed *ERF022* (KN and MDG—data not presented). This observation is consistent with the unmodified ethylene content and culture capacity for SE that was observed under *ERF022* overexpression. Altogether, the phenotypes of *erf022* mutant and *ERF022*-overexpressing cultures suggest that although *ERF022* activity is required for efficient SE induction (possibly via LEC2-mediated biosynthesis of auxin), other genetic elements that are interacting with *LEC2* and *ERF022* seem to determine the level of ethylene and auxin and the embryogenic response of explants.

A close link between auxin and ethylene in the mechanism that controls the embryogenic capacity in Arabidopsis was also suggested in an embryogenic callus culture (Bai et al. 2013). It was found that a depletion of auxin from the medium that results in the formation of somatic embryos is associated with a local *YUCCA* expression and a down-regulation of ethylene biosynthesis (Bai et al. 2013). However, it remains to be clarified whether *LEC2* of auxin-stimulated expression during the direct induction of SE (Ledwoń and Gaj 2009) is also involved in the *YUC*-mediated auxin biosynthesis that was observed in the embryogenic callus forced to produce somatic embryos on an auxin-free medium.

## Conclusions

The complex interactions between auxin, which has a prominent role in the induction of SE, and other hormones, including ethylene, are expected in the regulation of embryogenic responses in different plants, including Arabidopsis (Jimenez 2005; Bai et al. 2013). The results of this study provide novel hormone-related clues that can help to define the genetic network governing SE. The *ERF022*, a new ethylene regulator, was identified as controlling the embryogenic transition in the somatic cells of IZEs in Arabidopsis. To decipher the genes that play a crucial role in these interactions, the identification of the TFs that govern the primary crosstalk between auxin and ethylene is of great importance. The present study provides evidence that ERF022-LEC2 interaction may have a key role in the auxin–ethylene crosstalk that is associated with the induction of SE. Further analyses are needed to confirm the suggested genetic interactions between auxin- and ethylene-related genes and to identify other components of the regulatory circle that are proposed in the model (Fig. 12).

### *Author contribution*

MDG and KN conceived and designed research. KN and BW conducted the experiments. KN and MDG analysed the data. MDG and KN wrote the manuscript. All of the authors read and approved the manuscript.

## Electronic supplementary material

Below is the link to the electronic supplementary material.

Supplemental Fig. S1 Expression level of the *ERF022* gene in an IZE-culture on an E5 medium of SE-impaired mutants (*tan1-2*, *cbp20*, *lec2*). Relative transcript level was normalised to an internal control (*At4g27090*) and calibrated to Col-0 culture of the same age. * Values significantly different from Col-0 culture of the same age (*P* < 0.05; *n* = 3 ± SD) (TIFF 311 kb)

Supplemental Fig. S2 Expression level of the *ERF022* gene under ACC treatment. Relative transcript level was normalised to an internal control (*At4g27090*) and calibrated to the control treatment. * Values significantly different from control (*P* < 0.05; *n* = 3 ± SD) (TIFF 43 kb)

Supplemental Fig. S3 High efficiency (**a**) and productivity (**b**) of shoot ORG in the IZE explant culture of Col-0, pER8-ERF022 and *erf022*. *ERF022* overexpression was induced with ß-estradiol (+E). *n* = 3 ± SD (TIFF 786 kb)

Supplemental Fig. S4 Expression analysis with semi-qPCR analysis of *ACS7*, *ERF1*, *ACS8*, *ERF5* (**a**) and *ETR1* (**b**) in the seedlings of Col-0, pER8-ERF022 transgenic line and *erf022* mutant. *ERF022* overexpression was induced with ß-estradiol (+E). M, size marker (TIFF 613 kb)

Supplemental Fig. S5 Phenotypes of seven-day old seedlings of Col-0, pER8-ERF022 and *erf022* in response to MeJA. MS medium was supplemented with 10 µM of MeJA. *ERF022* overexpression was induced with ß-estradiol (+E). *Scale bars*, 1 cm (TIFF 3155 kb)

Supplemental Fig. S6 Expression level of the *LEC2* gene in the IZE-derived culture that was induced on the control (E5) and the ACC supplemented medium. Relative transcript level was normalised to an internal control (*At4g27090*) and calibrated to 0d. * Values significantly different from the control at the same age (*P* < 0.05; *n* = 3 ± SD) (TIFF 108 kb)

## Abbreviations

2,4-D: 2,4-Dichlorophenoxyacetic acid
2iP: 6-(a,a-Dimethylallylamino)-purine
ACC: 1-Aminocyclopropane-1-carboxylic acid
AVG: Aminoethoxyvinylglycine
BAP: 6-Benzylaminopurine
CIM: Callus induction medium
Ct: Threshold cycle
E5: Embryogenesis induction medium with auxin
E0: Auxin-free induction medium
IAA: Indole-3-acetic acid
IZE: Immature zygotic embryo
JA: Jasmonic acid
MeJA: Methyl jasmonate
NAA: 1-Naphthaleneacetic acid
ORG: Shoot organogenesis
SE: Somatic embryogenesis
SIM: Shoot induction medium
TF: Transcription factor
