# Supplementary figures and images for: ERF022 impacts the induction of somatic embryogenesis in Arabidopsis through the ethylene-related pathway
Source: Planta. 2014 Dec 23;241(4):967–85. doi: 10.1007/s00425-014-2225-9 (PMC4361773; doi:10.1007/s00425-014-2225-9)

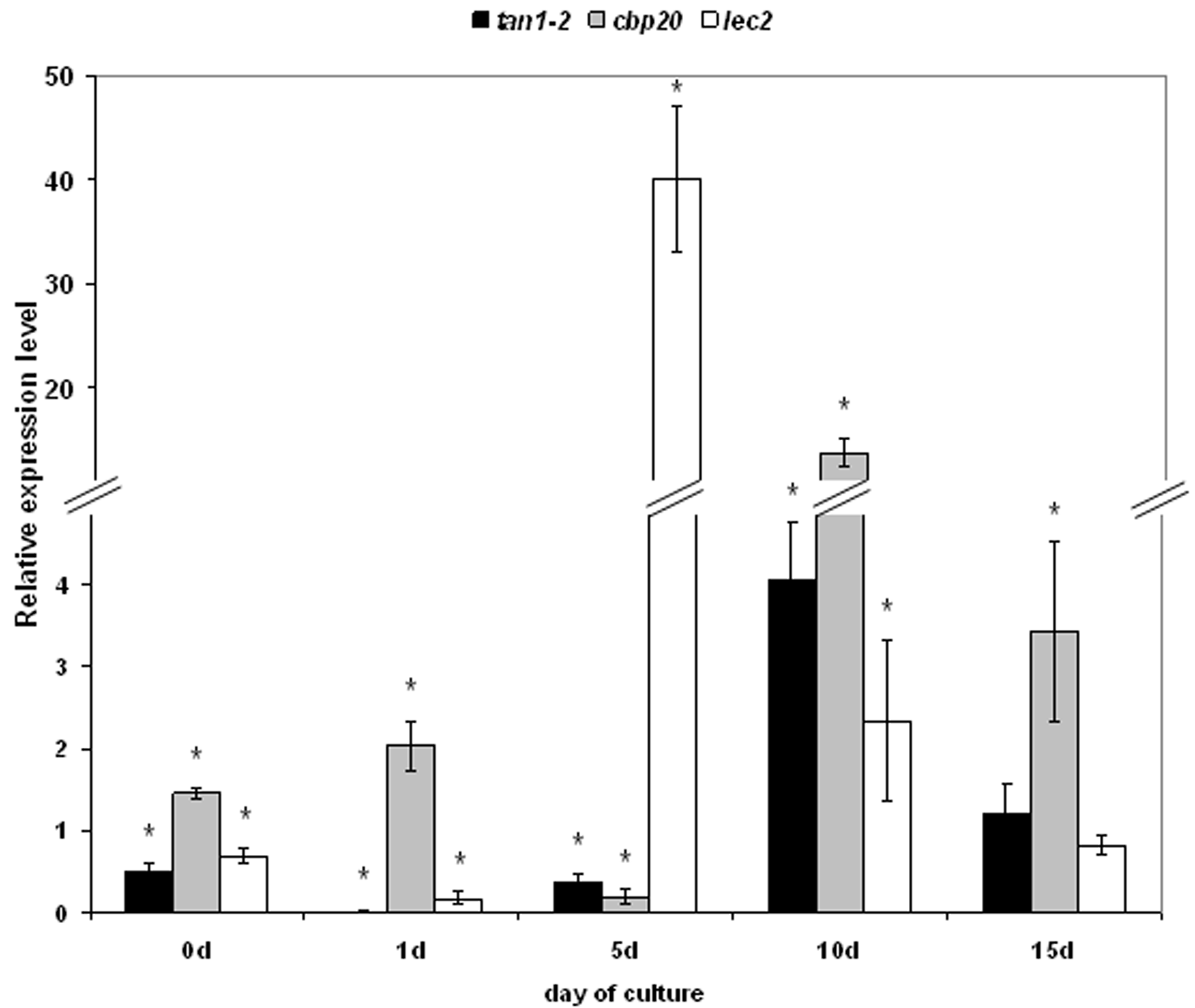

Supplement: Supplementary file 1 — Supplemental Fig. S1 Expression level of the ERF022 gene in an IZE-culture on an E5 medium of SE-impaired mutants (tan1-2, cbp20, lec2). Relative transcript level was normalised to an internal control (At4g27090) and calibrated to Col-0 culture of the same age. * Values significantly different from Col-0 culture of the same age (P < 0.05; n = 3 ± SD) (TIFF 311 kb) [file 425_2014_2225_MOESM1_ESM.tif]

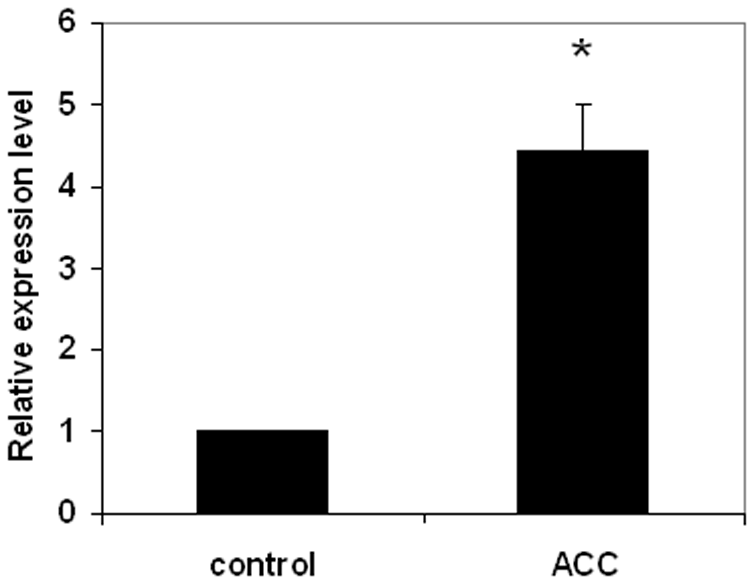

Supplement: Supplementary file 2 — Supplemental Fig. S2 Expression level of the ERF022 gene under ACC treatment. Relative transcript level was normalised to an internal control (At4g27090) and calibrated to the control treatment. * Values significantly different from control (P < 0.05; n = 3 ± SD) (TIFF 43 kb) [file 425_2014_2225_MOESM2_ESM.tif]

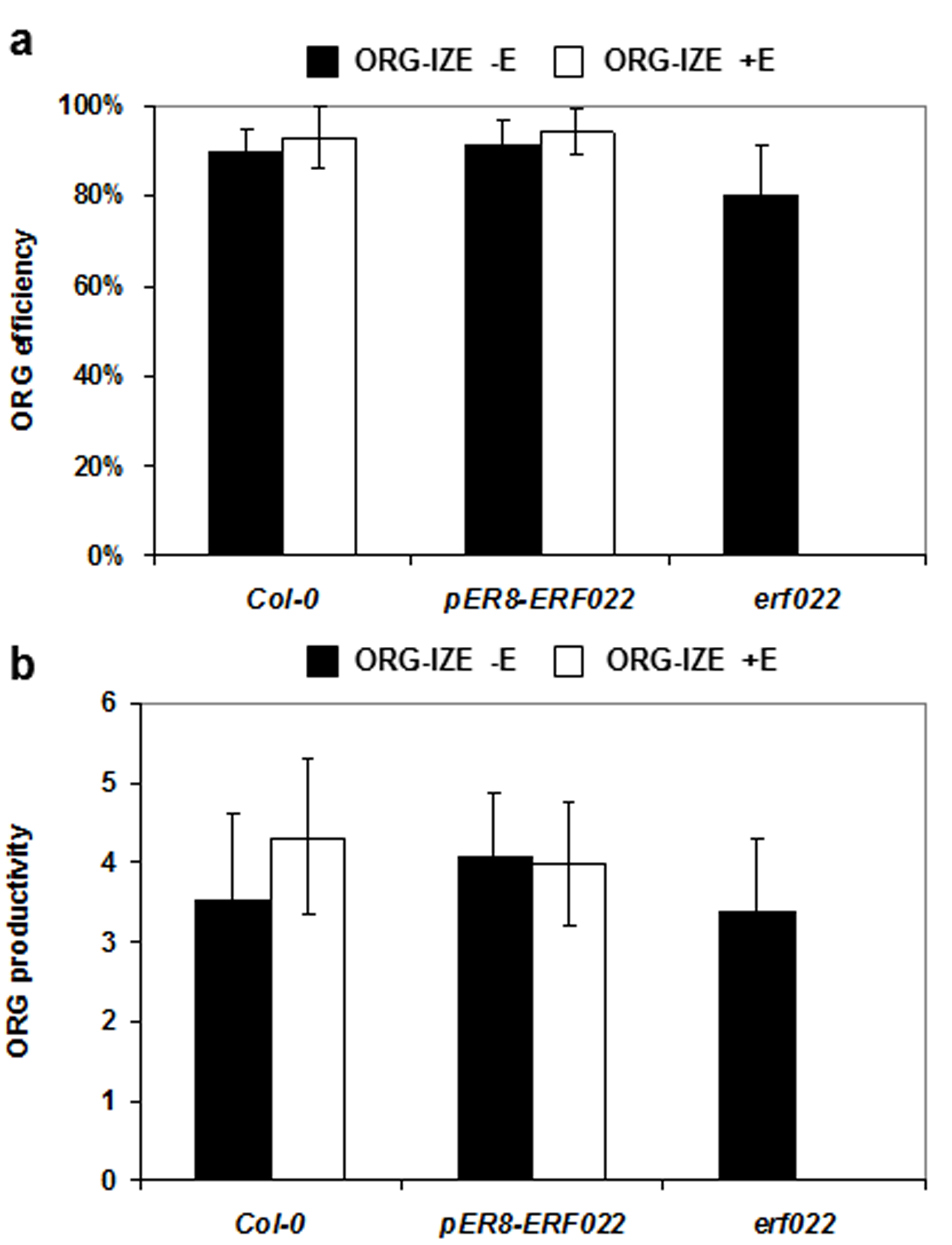

Supplement: Supplementary file 3 — Supplemental Fig. S3 High efficiency (a) and productivity (b) of shoot ORG in the IZE explant culture of Col-0, pER8-ERF022 and erf022. ERF022 overexpression was induced with ß-estradiol (+E). n = 3 ± SD (TIFF 786 kb) [file 425_2014_2225_MOESM3_ESM.tif]

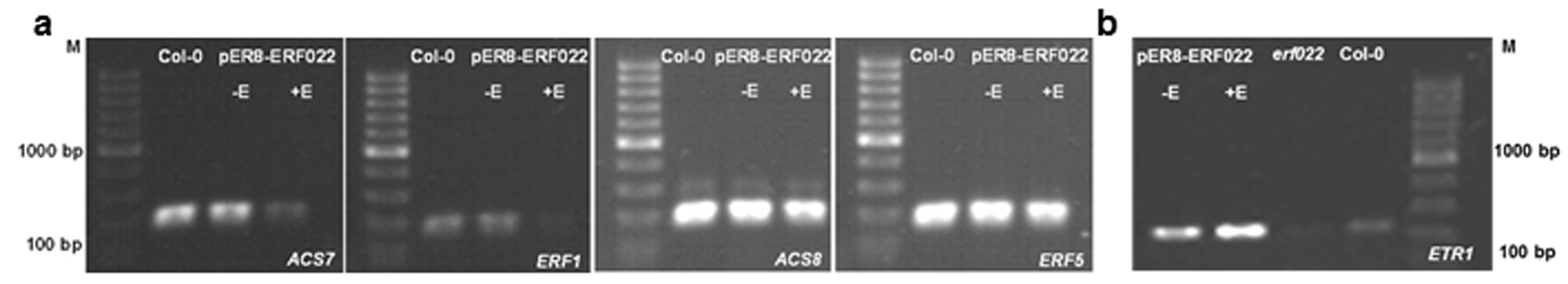

Supplement: Supplementary file 4 — Supplemental Fig. S4 Expression analysis with semi-qPCR analysis of ACS7, ERF1, ACS8, ERF5 (a) and ETR1 (b) in the seedlings of Col-0, pER8-ERF022 transgenic line and erf022 mutant. ERF022 overexpression was induced with ß-estradiol (+E). M, size marker (TIFF 613 kb) [file 425_2014_2225_MOESM4_ESM.tif]

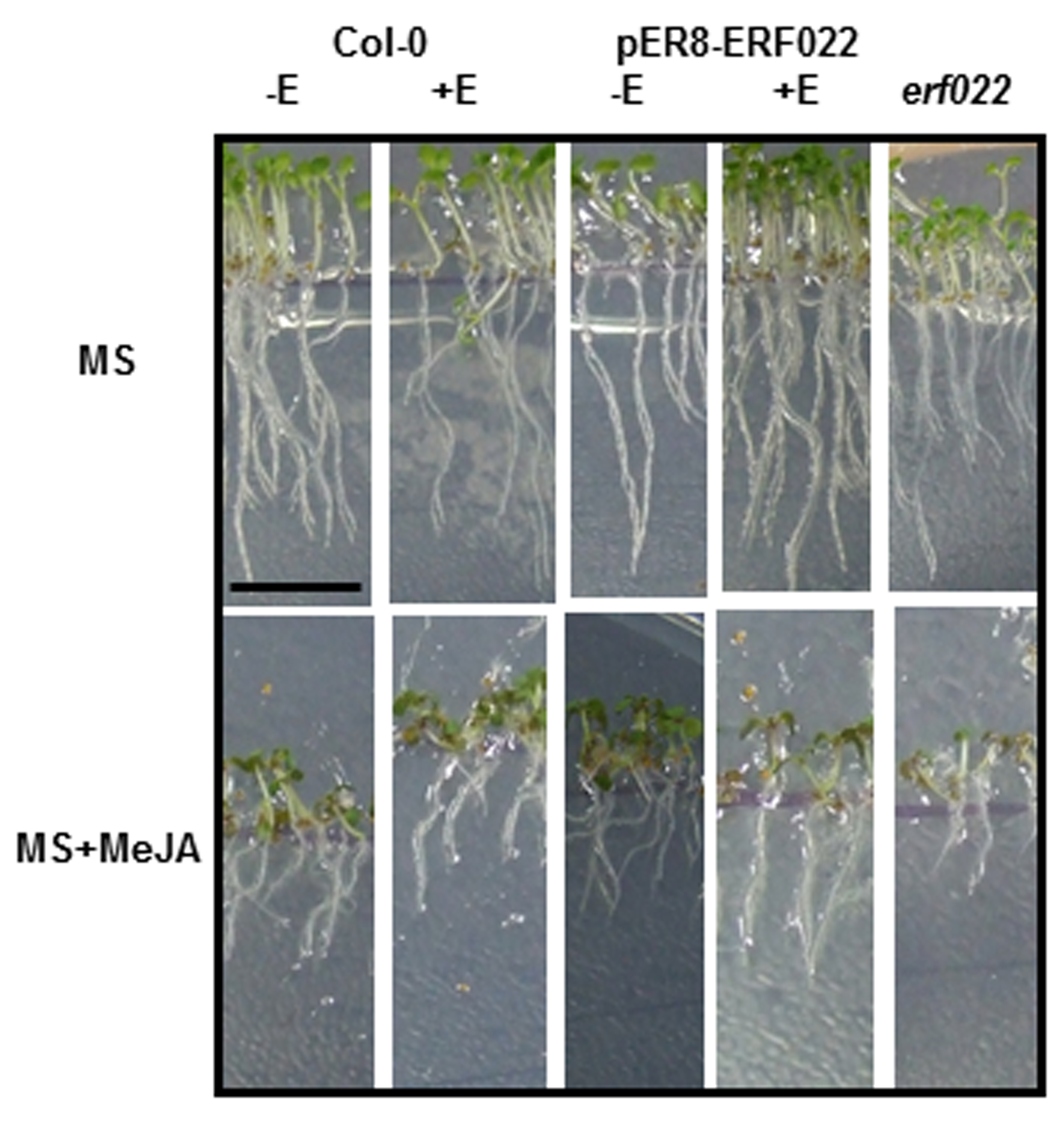

Supplement: Supplementary file 5 — Supplemental Fig. S5 Phenotypes of seven-day old seedlings of Col-0, pER8-ERF022 and erf022 in response to MeJA. MS medium was supplemented with 10 µM of MeJA. ERF022 overexpression was induced with ß-estradiol (+E). Scale bars, 1 cm (TIFF 3155 kb) [file 425_2014_2225_MOESM5_ESM.tif]

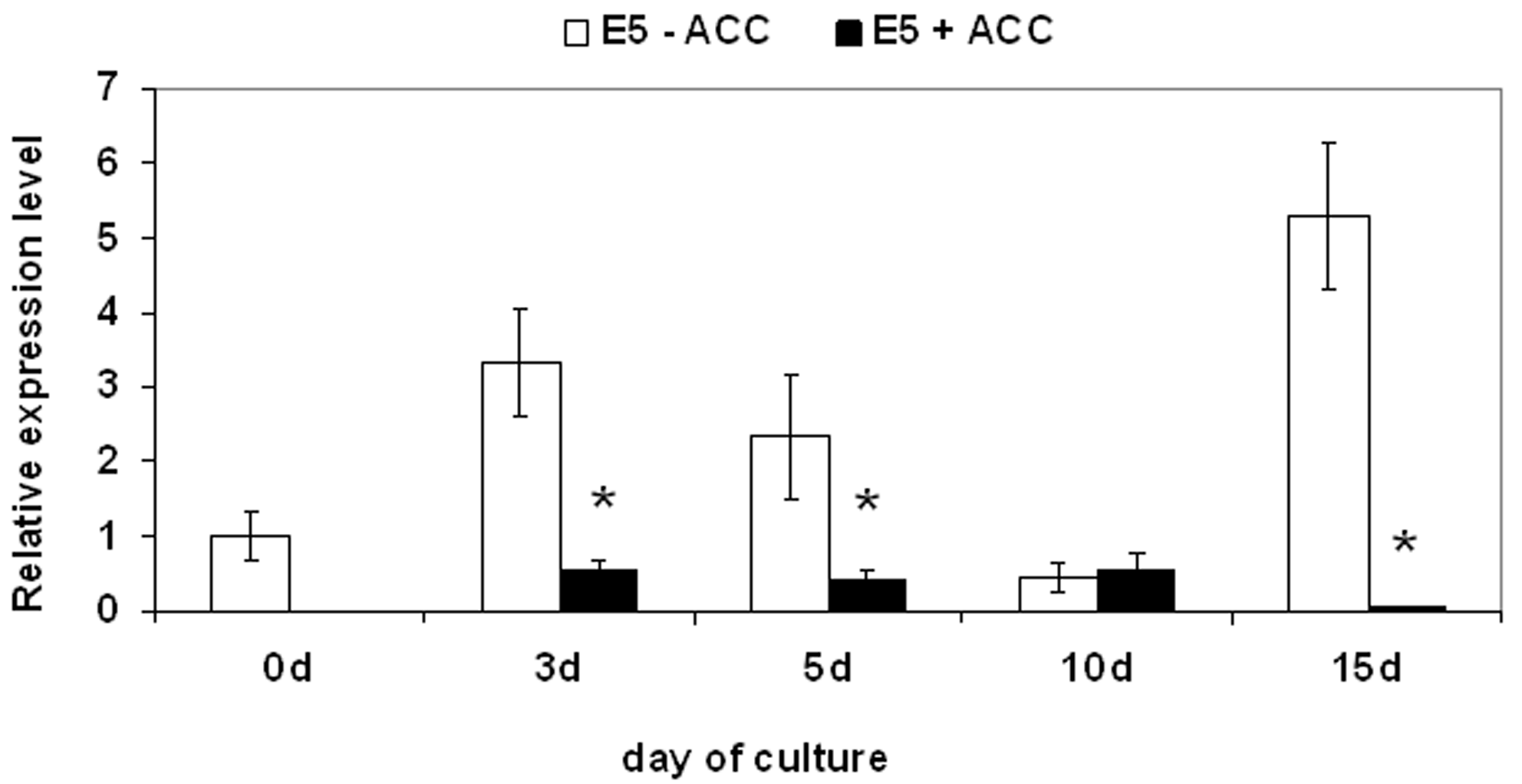

Supplement: Supplementary file 6 — Supplemental Fig. S6 Expression level of the LEC2 gene in the IZE-derived culture that was induced on the control (E5) and the ACC supplemented medium. Relative transcript level was normalised to an internal control (At4g27090) and calibrated to 0d. * Values significantly different from the control at the same age (P < 0.05; n = 3 ± SD) (TIFF 108 kb) [file 425_2014_2225_MOESM6_ESM.tif]
